# Rare deleterious germline variants and risk of lung cancer

**DOI:** 10.1038/s41698-021-00146-7

**Published:** 2021-02-16

**Authors:** Yanhong Liu, Jun Xia, James McKay, Spiridon Tsavachidis, Xiangjun Xiao, Margaret R. Spitz, Chao Cheng, Jinyoung Byun, Wei Hong, Yafang Li, Dakai Zhu, Zhuoyi Song, Susan M. Rosenberg, Michael E. Scheurer, Farrah Kheradmand, Claudio W. Pikielny, Christine M. Lusk, Ann G. Schwartz, Ignacio I. Wistuba, Michael H. Cho, Edwin K. Silverman, Joan Bailey-Wilson, Susan M. Pinney, Marshall Anderson, Elena Kupert, Colette Gaba, Diptasri Mandal, Ming You, Mariza de Andrade, Ping Yang, Triantafillos Liloglou, Michael P. A. Davies, Jolanta Lissowska, Beata Swiatkowska, David Zaridze, Anush Mukeria, Vladimir Janout, Ivana Holcatova, Dana Mates, Jelena Stojsic, Ghislaine Scelo, Paul Brennan, Geoffrey Liu, John K. Field, Rayjean J. Hung, David C. Christiani, Christopher I. Amos

**Affiliations:** 1 Dan L. Duncan Comprehensive Cancer Center, Department of Medicine, Baylor College of Medicine, Houston, TX USA; 2 Institute for Clinical and Translational Research, Baylor College of Medicine, Houston, TX USA; 3 International Agency for Research on Cancer, Lyon, France; 4 Department of Molecular and Human Genetics, Baylor College of Medicine, Houston, TX USA; 5 Department of Pediatrics, Baylor College of Medicine, Houston, TX USA; 6 Michael E. DeBakey Veterans Affairs Medical Center, Houston, TX USA; 7 Department of Biomedical Data Science, Geisel School of Medicine, Dartmouth College, Lebanon, NH USA; 8 Karmanos Cancer Institute, Wayne State University, Detroit, MI USA; 9 Department of Translational Molecular Pathology, The University of Texas MD Anderson Cancer Center, Houston, TX USA; 10 Channing Division of Network Medicine, Department of Medicine, Brigham and Women’s Hospital and Harvard Medical School, Boston, MA USA; 11 National Human Genome Research Institute, Bethesda, MD USA; 12 University of Cincinnati College of Medicine, Cincinnati, OH USA; 13 The University of Toledo College of Medicine, Toledo, OH USA; 14 Louisiana State University Health Sciences Center, New Orleans, LA USA; 15 Medical College of Wisconsin, Milwaukee, WI USA; 16 Mayo Clinic College of Medicine, Rochester, MN USA; 17 Mayo Clinic College of Medicine, Scottsdale, AZ USA; 18 Roy Castle Lung Cancer Research Programme, The University of Liverpool, Department of Molecular and Clinical Cancer Medicine, Liverpool, UK; 19 M. Sklodowska-Curie National Research Institute of Oncology, Warsaw, Poland; 20 Nofer Institute of Occupational Medicine, Department of Environmental Epidemiology, Lodz, Poland; 21 Russian N.N. Blokhin Cancer Research Centre, Moscow, Russian Federation; 22 Faculty of Health Sciences, Palacky University, Olomouc, Czech Republic; 23 Institute of Public Health and Preventive Medicine, Charles University, 2nd Faculty of Medicine, Prague, Czech Republic; 24 National Institute of Public Health, Bucharest, Romania; 25 Department of Thoracopulmonary Pathology, Service of Pathology, Clinical Center of Serbia, Belgrade, Serbia; 26 Princess Margaret Cancer Center, Toronto, ON Canada; 27 Lunenfeld-Tanenbaum Research Institute, Sinai Health System, Toronto, ON Canada; 28 Harvard University T. H. Chan School of Public Health, Boston, MA USA

**Keywords:** Non-small-cell lung cancer, Cancer epidemiology

## Abstract

Recent studies suggest that rare variants exhibit stronger effect sizes and might play a crucial role in the etiology of lung cancers (LC). Whole exome plus targeted sequencing of germline DNA was performed on 1045 LC cases and 885 controls in the discovery set. To unveil the inherited causal variants, we focused on rare and predicted deleterious variants and small indels enriched in cases or controls. Promising candidates were further validated in a series of 26,803 LCs and 555,107 controls. During discovery, we identified 25 rare deleterious variants associated with LC susceptibility, including 13 reported in ClinVar. Of the five validated candidates, we discovered two pathogenic variants in known LC susceptibility loci, *ATM* p.V2716A (Odds Ratio [OR] 19.55, 95%CI 5.04–75.6) and *MPZL2* p.I24M frameshift deletion (OR 3.88, 95%CI 1.71–8.8); and three in novel LC susceptibility genes, *POMC* c.*28delT at 3′ UTR (OR 4.33, 95%CI 2.03–9.24), *STAU2* p.N364M frameshift deletion (OR 4.48, 95%CI 1.73–11.55), and *MLNR* p.Q334V frameshift deletion (OR 2.69, 95%CI 1.33–5.43). The potential cancer-promoting role of selected candidate genes and variants was further supported by endogenous DNA damage assays. Our analyses led to the identification of new rare deleterious variants with LC susceptibility. However, in-depth mechanistic studies are still needed to evaluate the pathogenic effects of these specific alleles.

## Introduction

Lung cancer (LC), the leading cause of cancer mortality in the US, has recently shown substantial drops in mortality, largely attributed to reduced smoking rates and improvement in new treatments such as immunotherapy^1^. Prior genome-wide association studies (GWAS) identified novel genetic factors influencing LC risk, which are sometimes modulated by smoking behavior^2^. Notably, in the 15q25.1 region that shows the most significant and consistent genetic signal, a missense p.D398N and a 22-bp deletion (del) in the core promoter region of *CHRNA5* have been identified that affect the function and expression^3,4^. Carriers of these variants find quitting smoking more difficult than noncarriers^5^ and may benefit from a targeted smoking cessation intervention^6^.

Previous studies have estimated heritability of LC to be 18%^7^. Recent genetic studies suggest that rare variants (minor allele frequency [MAF] < 1%) that are functionally deleterious, exhibit far larger effect sizes than common variants^8–10^ as they display signs of stronger selective pressure^11,12^, and could account for missing heritability unexplained by common variants^11^. Fewer than 3% of protein-coding single nucleotide variants (SNVs) corresponding to approximately 300 genes per genome are predicted to result in loss of protein function (LoF) through the introduction of stop-gain, frameshift, or the disruption of an essential splice site^13^. Insertions (ins) or deletions (indels) have been understudied, though they are the second most abundant source of human genetic variation. Selected indels have been identified as playing a key role in causing LC, such as p.E746_A750del in *EGFR*^14–16^.

Supporting the hypothesis that deleterious mutations will show lower MAF are recent identifications of several rare missense variants that have a moderate-to-large effect on LC risk, for example, *PARK2* p.R275W (OR 5.24)^17^, *BRCA2* p.K3326X (OR 2.47), *CHEK2* p.I157T (OR 0.38)^18^, *LTB* p.L87F (OR 7.52), *P3H2* p.Q185H (OR 5.39)^19^, *DBH* p.V26M^20^, and *ATM* p.L2307F (OR 8.82)^21^. Because of the stronger evolutionary pressure and weak linkage disequilibrium (LD) with common SNPs used in GWAS, finding these rare variants through population-based studies can be challenging^22^. To maximize the potential for the detection of large-effect, rare deleterious variants (SNVs and small indels ≤21 bp), we employed whole exome sequencing (WES) plus targeted sequencing on healthy controls and selected high-risk LC cases enriched with the highest genetic risk of LC, for example, early-onset or family history of LC (FHLC)^7,23,24^.

## Results

### Demographics of study subjects

As shown in Table 1, the vast majority of subjects in the discovery study ─ Transdisciplinary Research in Cancer of the Lung (TRICL; 1,045 LCs vs. 885 controls) ─ and the validation sets (26,803 LCs and 555,107 controls) were primarily of European-descent (Supplementary Fig. 1). LC cases were significantly more likely to be smokers and with higher pack-years than controls (*P*-value < 0.0001). The TRICL and Genetic Epidemiology of LC Consortium (GELCC) cases were enriched for having FHLC.

**Table 1:** Basic characteristics of LC cases and controls in the discovery and validations sets.

	Discovery		Validation^#^									
Characteristics	TRICL		GELCC	COPDGene	TCGA	gnomAD	OncoArray		Affymetrix		UKB	
Platform	WES		WES	WES	WES	WES + WGS	Genotyping		Exome array		Genotyping	
*N* (%)^&^	LC Case *n* = 1045	Control *n* = 885	LC case *n* = 380	Controls *n* = 318	LC cases *n* = 1015	Controls *n* = 134,187	LC cases *n* = 17,878	Controls *n* = 13,425	LC case *n* = 5364	Controls *n* = 5724	LC Case *n* = 2166	Controls *n* = 401,453
Ethnicity		*P* < 0.0001				*P* < 0.0001						
White	909 (87%)	830 (94%)	372 (98%)	318 (100%)	742 (73%)	94,134 (70%)	13,876 (78%)	11,011 (82%)	3086 (58%)	3550 (62%)	2094 (97%)	375,894 (94%)
Other†	136 (13%)	55 (6%)	6 (2%)	0	273 (27%)	40, 053 (30%)	210 (1%)	128 (1%)	625 (12%)	652 (11%)	65 (3%)	24,055 (6%)
Age, yr.		*P* = 0.006										*P* < 0.0001
Mean (range)	63 (24–91)	61 (20–90)	64 (30–87)	63 (55–80)	65 (30–90)	54 (18–90)	64 (19–95)	62 (18–97)	61 (30–95)	59 (31–91)	62 (40–70)	56 (37–73)
<60 yr.	418 (40%)	356 (40%)	102 (27%)	88 (28%)	214 (21%)	–	6036 (43%)	5303 (40%)	2335 (43%)	3063 (53%)	624 (29%)	242,687 (60%)
Sex												*P* < 0.0001
Male	614 (59%)	515 (58%)	232 (61%)	172 (54%)	563 (59%)	73,370 (55%)	11,147 (62%)	8274 (62%)	2930 (55%)	3125 (55%)	1182 (55%)	186,083 (46%)
Female	431 (41%)	370 (42%)	171 (45%)	146 (46%)	452 (41%)	60,817 (45%)	6731 (38%)	5151 (38%)	2434 (45%)	2599 (45%)	984 (45%)	215,370 (54%)
Smoking		*P* < 0.0001						*P* < 0.0001		*P* < 0.0001		*P* < 0.0001
Never	125 (12%)	308 (35%)	31 (8%)	0	173 (17%)	–	1720 (10%)	4152 (31%)	572 (11%)	1726 (30%)	203 (10%)	236,246 (59%)
Ever	918 (88%)	576 (65%)	346 (91%)	318 (100%)	742 (73%)	–	15,889 (89%)	8998 (67%)	4675 (87%)	3972 (69%)	1945 (90%)	163,226 (41%)
Mean PY (range)	42 (0–196)	23 (0–133)	46 (0–165)	54 (10–97)	42 (0–154)	–	46(0–315)	33 (0–260)	45 (0–231)	34 (0–218)	40 (0–220)	23 (0–301)
FHLC		*P* < 0.0001										*P* < 0.0001
Yes	506 (48%)	72 (8%)	122 (33%)	–	–	–	–	–		–	457 (21%)	49,104 (12%)
No	359 (34%)	306 (35%)	258 (67%)	–	–	–	–	–		–	1709 (79%)	352,349 (88%)
Histology												
AD	459 (44%)	–	182 (48%)	–	577 (57%)	–	6568 (37%)	–	2106 (39%)	–	781 (36%)	–
SCC	342 (33%)	–	118 (31%)	–	438 (43%)	–	4284 (24%)	–	1131 (21%)	–	461 (21%)	–
Other	244 (23%)	–	80 (21%)	–	0	–	7026 (39%)	–	2127 (40%)	–	924 (43%)	–

*TRICL* Transdisciplinary Research in Cancer of the Lung, *WES* whole-exome sequencing, *WGS* whole-genome sequencing, *LC* lung cancer, *PY* pack-year, *FHLC* family history of LC (first degree), *AD* adenocarcinoma, *SCC* squamous cell carcinoma.

^*&*^Numbers do not add up due to missing data.

†Other ethnicities in TRICL (one African control subject and 190 unknown), TCGA (8% African American 2% East Asian, and 17% unknown), gnomAD (8.8% African, 7.2% East Asian, 11.4% South Asian, and 2.5% other). Genetic ancestry analysis of TRICL subjects shows most of the subjects of the “unknown” race were located between the European- and Asian-ancestry clusters (Supplemental Fig. 1). Genetic ancestry analysis of TCGA patients shows the vast majority of subjects with “unknown” race were primarily genetic European ancestry (i.e., 90% TCGA-LCs were genetically Europeans)^82^.

#The validation sets include 26,803 LCs and 555,107 controls: (1) Genetic Epidemiology of LC (GELCC) WES data for 380 LCs (258 sporadic and 122 FLC were selected from high-risk LC families with at least two first-degree relatives affected with LC); (2) COPDGene WES data for 318 controls with normal lung function; (3) TCGA (The Cancer Genome Atlas) germline WES data for 1015 LCs; (4) GnomAD (genome aggregation database, v2.1) WES and WGS data for 134,187 non-cancer controls (excluded individuals from cancer cohort studies, such as the TCGA cohort). (5) OncoArray genotyping data for 17,878 LCs vs. 13,425 controls; (6) Affymetrix exome array data for 5364 LCs vs. 5724 controls; (7) UK Biobank (UKB) genotyping data for 2166 LCs vs. 401,453 controls.

### Identification of rare and deleterious variants in the TRICL discovery set

In the discovery set, a total of 2,182,753 variants were detected. Applying a three-step filtering method based on allele frequency (MAF < 1% in non-Finnish European [NFE] population from the Genome Aggregation Database [gnomAD]), variant class (missense, protein-truncating and regulatory), and functional effects (predicted deleterious and or with clinical significance from ClinVar), we identified 67,470 rare and putatively deleterious variants: 63% missense, 16% frameshift (fs), 12% in-frame indels, 6% regulatory (untranslated region [UTR] and splice acceptor/donor), and 3% stop-gain. Single variant association analysis identified 75 potential candidates.

Given the known challenge of excessive false-positive indel detection rates caused by the high frequency of homopolymer-associated sequencing errors^25–28^, we subjected these 75 potential candidates to additional filtering and manual inspection using Genome Browser (Supplementary Table 1). Twenty-five of the 75 were high-confidence putative candidates (two SNVs, four ins, and 19 del). Supplementary Fig. 2 shows the variant visualization map for the candidates and variant carriers (read alignment and depth). Thirteen out of the 25 candidates (in 24 genes) reported clinical significance in ClinVar, and eight were classified as pathogenic. Also, 5/24 genes were mapped to known LC-GWAS loci, such as 3q28 *TP63*^29^, 5q31.1 *TXNDC15*^30^, 11q22.3 *ATM*^21^, 11q23.3 *MPZL2*^31^, and 22q12.1 *CHEK2*^18^. Three mapped in known GWAS loci for COPD/ PF (pulmonary function): 1p34.3 *BMP8A*^32,33^, 1p36.31 *PHF13*^32^, and 14q23.1 *TALPID3/KIAA0586*^34^.

We next assessed the dose-effect of the 25 candidates: 16 were enriched in LCs (risk-conferring alleles) and 9 were enriched in controls (protective alleles). Compared with subjects with zero risk- and protective-alleles, the groups carrying one, and two risk-alleles (5 LCs) showed a progressively increased risk, whereas groups carry one, and two protective-alleles (6 controls) demonstrated a gradually reduced risk (Supplementary Table 2). All 6 controls harbored *MOB3A* p.F69_I75del, whereas 4/5 LCs harbored *STAU2* p.N364M fs*67del.

Studying the demographics of the mutation carriers, there was no significant difference in smoking (status and pack-years) or FHLC between carriers and non-carriers. Notably, 5/6 two-protective-alleles carriers were male, whereas 4/5 two-risk-alleles carriers were female and had adenocarcinoma (AD). Overall, age did not differ significantly between carriers and non-carriers (Supplementary Fig. 3). However, in LC cases, onset-age in risk-allele carriers (54 yrs for two-risk-alleles carriers, 62 yrs for one-risk-allele carriers) were significantly younger than the protective-allele carriers (69 yrs; Supplementary Table 2).

Further gene-environment (G×E) interaction analysis showed that two variants interacted with smoking behavior (Supplementary Table 1). Specifically, the risk *MLNR* p.Q334V fs*3del interacted with pack-years (*P*-value 0.0035); the protective-effect associated with the *MOB3A* p.F69_I75del is substantial and significant among males *(*10/11 control carriers were male, whereas 0/2 LCs carriers were male; *P*-value 0.042), smokers (6/11 control carriers were smokers, whereas 0/2 LCs carriers were smokers; *P*-value 0.016), and pack-years (*P*-value 0.0036). We also identified that the protective variant *TXNDC15* p.E9G fs*68del interacted with FHLC, as 5/7 of LC carriers with FHLC, compared to 0/21 controls (*P*-value 0.035).

We subsequently conducted gene-based rare variant burden tests for the 24 genes harboring potential candidates, five genes, namely, *MLNR*, *CCDC105*, *BMP8A*, *MME*, and *NPHP3*, showed suggestive associations (Table 2). We also performed exome wide gene-based tests, however, none showed strong association after multiple testing corrections (Supplementary Fig. 4).

**Table 2:** Gene-based association tests in the TRICL study, ranked by *P*-value from the combined multivariate and collapsing test.

Genes	*N*. rare deleterious variants*	*N*. multi-marker genotypes	*N*. carriers LC /Control	KBAC test *P*-value	CMC test *P*-value	CMC test OR (95% CI)	Gene constraint LoF o/e (90% CI)^&^	Gene PhoRank to phenotype^#^
Risk genes								
CCDC105	20	11	28/5	**0.012**	**0.013**	5.63 (0.87–31.4)	0.71 (0.46–1.12)	0.12 _ PF
BMP8A	3	4	11/2	**0.014**	**0.014**	4.22 (1.14–36.3)	0.8 (0.49–1.35)	0.32 _ PF
MME/CD10	5	6	7/0	**0.014**	**0.015**	1.85 (0.65–11.16)	0.7 (0.54–0.92)	**0.83 _ LC**
NPHP3	6	7	7/0	**0.015**	**0.015**	1.68 (0.76–15.4)	0.5 (0.38–0.65)	0.68 _ PF
MLNR	5	6	11/2	**0.005**	**0.022**	4.19 (1.12–38.9)	0.51 (0.5–1.16)	0.09 _ PF
NKX6-1	9	11	47/31	0.064	0.048	1.30 (0.82–2.06)	0.39 (0.18–1.0)	0.28 _ LC
ENAM	7	8	9/2	0.043	0.065	2.94 (0.79–9.07)	0.6 (0.44–0.84)^`^	0.32 _ PF
ATM	15	15	16/11	0.591	0.098	1.58 (0.69–7.04)	0.60 (0.51–0.71)	**0.95 _ LC**
RHBDD3	9	10	11/4	0.101	0.102	1.64 (0.85–23.6)	0.71 (0.41–1.27)	0.16 _ PF
STAU2	27	31	107/76	0.141	0.213	1.21 (0.89–1.65)	**0.14 (0.07–0.32)**	0.23 _ LC
TALPID3	11	12	17/8	0.139	0.403	1.64 (0.93–2.24)	0.54 (0.42–0.72)	0.61 _ PF
MPZL2	6	7	7/3	0.153	0.403	1.34 (0.77–2.13)	1.34 (0.9–1.86)	0.12 _ LC
TP63	6	7	9/3	0.396	0.539	1.20 (0.59–12.3)	**0.13 (0.07–0.27)**	**0.87 _ LC**
POMC	6	7	7/5	0.744	0.790	1.45 (0.57–3.71)	0.74 (0.42–1.38)	0.30 _ PF
F13B	5	6	7/5	0.965	0.905	1.02 (0.81–1.27)	0.59 (0.41–0.85)	0.34 _ PF
Protective genes								
TXNDC15	11	12	10/27	0.746	0.001	0.31 (0.15–0.64)	0.38 (0.21–0.76)	0.60 _ PF
GJB6	2	3	0/6	0.877	0.008	0.12 (0.02–0.66)	1.07 (0.66–1.74)	0.31 _ LC
MOB3A	3	4	3/12	0.587	0.008	0.21 (0.05–0.67)	0.96 (0.54–1.7)	0.10 _ LC
CASQ2	2	4	15/26	0.955	0.013	0.73 (0.44–1.23)	0.94 (0.65–1.38)	0.36 _ PF
OR51J1	2	3	1/6	0.351	0.037	0.14 (0.03–0.84)	0.19 (0.07–0.88)	0.10 _ PF
FAM111A	5	6	11/21	0.406	0.076	0.42 (0.20–0.92)	2.09 (0.66–1.95)	0.29 _ LC
PHF13	9	10	16/12	0.097	0.742	1.23 (0.53–5.17)	**0.01 (0–0.25)**	0.28 _ LC + PF
MLKL	17	19	30/28	0.055	0.689	0.95 (0.54–3.52)	0.87 (0.63–1.24)	0.20 _ PF
CHEK2	8	9	7/5	0.484	0.811	1.11 (0.64–2.02)	1.15 (0.87–1.53)	**0.97 _ LC**

*TRICL* Transdisciplinary Research in Cancer of the Lung, *CMC* Combined Multivariate and Collapsing, *KBAC* Kernel-Based Adaptive Cluster, *LoF* loss of function, *LC* lung cancer, *PF* pulmonary function, *OR* odds ratio, *CI* confidence interval, *o/e* observed/expected.

*Number of rare deleterious variants within the genes. False discovery rate (FDR) adjusted *P*-value was reported.

^&^Gene constraint LoF o/e values developed with gnomAD: observed counts are based on sequencing data from gnomAD, expected counts are based on a mutational model that takes sequence context and coverage into account. Lower o/e, in particular, the upper bound of the CI < 0.35 are indicative of strong intolerance (disease-causing). The top three genes with the lowest o/e were bolded: *PHF13*, *TP63*, and *STAU2*.

#Genes Phevo PhoRank is based on gene functions relevant to the disease phenotype (LC, COPD/PF) from diverse biomedical ontologies. Disease-associated genes have a higher Phevor score. The top four genes with the highest scores were bolded: *CHEK2*, *ATM*, *TP63*, and *MME/CD10*.

### Meta-analyses of the discovery and validation sets

In the seven validation datasets, of the 25 candidates, 100% were covered by the gnomAD, 22 (88%) in TCGA, 16 (64%) in COPDGene, nine (36%) in GELCC, and nine (36%) were covered in one of the three case–control studies (OncoArray, Affymetrix, and UKB) with genotyping data. Table 3 summarizes the top five candidates with consistent associations from the meta-analysis.

**Table 3:** Top five hits from discovery and validation association analysis.

Candidates	*n*. (freq.%) carriers				Meta-analysis^&^		
(ClinVar)	TRICL: 1045 LC, 885 Control	GELCC: 380 LC, COPDGene: 318 Control	TCGA: 1015 LC, GnomAD: 134,187 Control	GWAS studies @ OncoArray/Affymetrix	Total freq.% carrier LC/Control	OR (95% CI)	*P*-value
***ATM*** missense SNV p.V2716A, rs587782652 (Pathogenic)	2 (0.19%) / 0	0 / 0	2 (0.20%)/ 5 (0.004%)	5 (0.03%) / 0 @ OncoArray	0.05% / 0.003%	19.55 (5.04–75.6)	1.7e-05
***POMC*** 3′ UTR deletion c.*28delT, rs756770132 (VUS)	6 (0.57%) / 0	4 (1.05%) ^**1 FLC**^ / 0	6 (0.59%) / 207 (0.17%)	-	0.66% / 0.15%	4.33 (2.03–9.24)	0.00015
***STAU2*** LoF deletion p.N364M fs*67, rs746501298	21 (2.01%) / 4 (0.45%)	4 (1.05%) ^**3 FLC**^ / 0	0 / 25 (0.02%)	-	1.02% / 0.02%	4.48 (1.73–11.55)	0.0019
***MPZL2*** LoF deletion p.I24M fs*22, rs752672077 (Pathogenic)	3 (0.29%) / 0	4 (1.05%) ^**2 FLC**^ / 0	5 (0.49%) / 189 (0.15%)	-	0.49% / 0.14%	3.88 (1.71–8.8)	0.0012
***MLNR*** LoF deletion, p.Q334V fs*3, rs563947699	9 (0.86%) ^**3 FHLC**^/ 0	6 (1.58%) ^**2 FLC**^ / 0	7 (0.69%) / 431 (0.35%)	29 (0.54%) / 49 (0.86%) @ Affymetrix	0.65% / 0.34%	2.69 (1.33–5.43)	0.0060

*TRICL* Transdisciplinary Research in Cancer of the Lung, *SNV* single nucleotide variants, *Indels* insertion (ins)/deletion (del), *LoF* loss of function, *fs* frameshift, *VUS* variant of uncertain significance from ClinVar, *NA* not available, *LC* lung cancer, *AD* adenocarcinoma, *FLC* familiar lung cancer, *OR* odds ratio, *CI* confidence interval.

#The validation sets include 26,803 LCs and 555,107 controls: 1) Genetic Epidemiology of LC (GELCC) WES data for 380 LCs (122 FLC and 258 sporadic); 2) COPDGene WES data for 318 controls; 3) TCGA (The Cancer Genome Atlas) WES data for 1015 LCs; 4) GnomAD (genome aggregation database) WES and WGS data for 134,187 non-cancer controls; 5) OncoArray genotyping data for 17,878 LCs vs. 13,425 controls; 6) Affymetrix exome array data for 5364 LCs vs. 5724 controls; 7) UK Biobank (UKB) genotyping data for 2166 LCs vs. 401,453 controls.

^@^The *ATM* p.V2716A genotype was from the OncoArray study; the *MLNR* p.Q334V deletion genotype was from the Affymetrix study.

^&^The freq.% of carriers were based on the available cases and controls. False discovery rate (FDR) adjusted *P*-values indicate significant associations based on a fixed-effect meta-analysis.

**Fig. 1:**
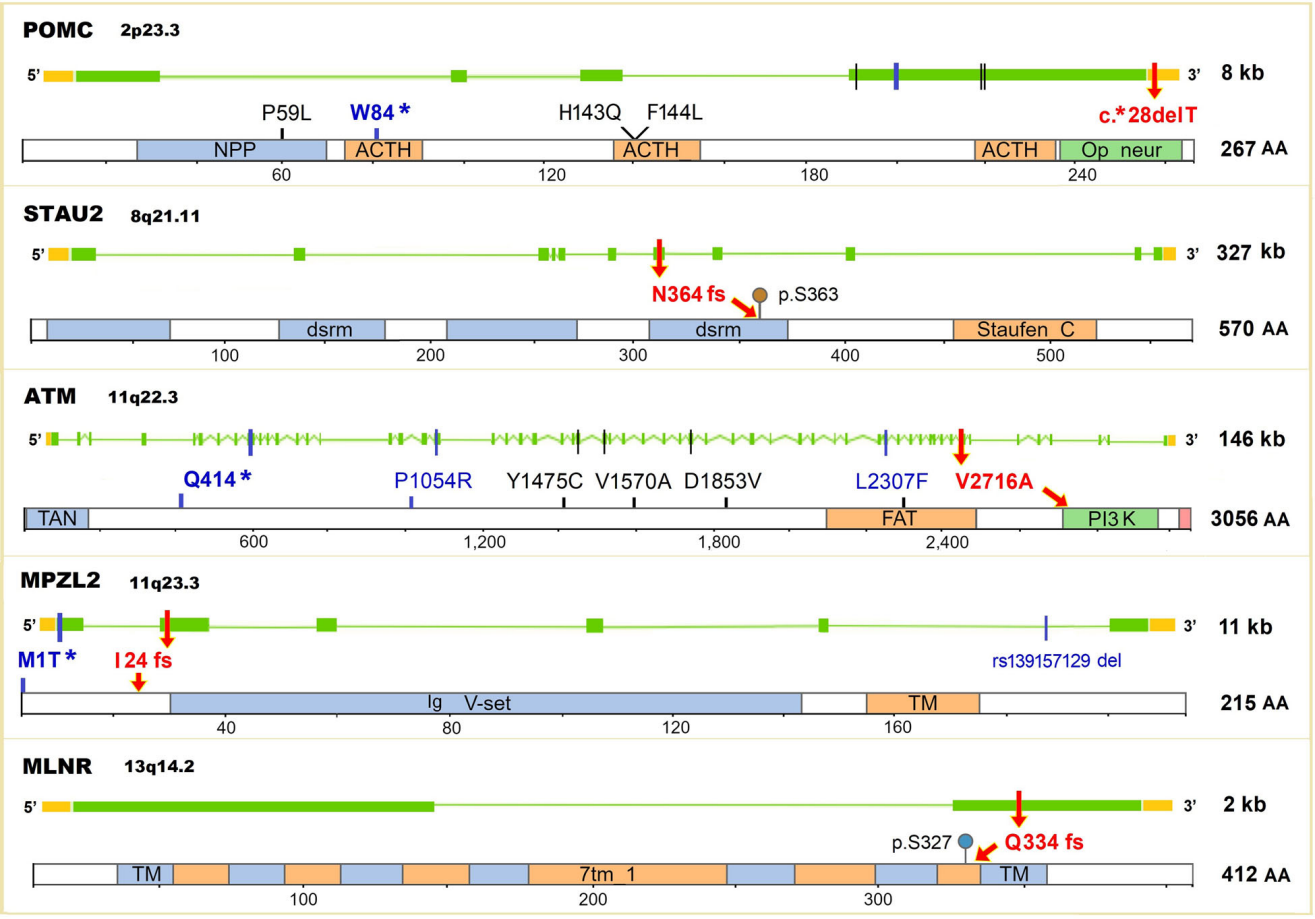
Gene exons, protein domains, and rare deleterious variants of the candidate genes. The top five candidate variants (red arrows): 1) *POMC* c.*28 deletion (del) located at target sites of several miRNAs in 3′ UTR; 2) *STAU2* p.N364M fs*67del located in the double-stranded RNA-binding motif (dsrm), and next to a phosphorylation site p.S363; 3) *ATM* V2716A located in the PI3-kinase (PI3K) catalytic domain; 4) *MPZL2* p.I24M fs*22del was close to the antibody variable domain of immunoglobulins (Ig-V); 5) *MLNR* p.Q334V fs*3del located in the transmembrane receptor domain (TM), and close to a phosphorylation site p.S327. The color vertical bars represent different types of variants: ClinVar pathogenic variants (bold blue: *POMC* W84* stop-gain, *ATM* Q414* stop-gain, and *MPZL2* M1T* start-loss), previous reported LC-associated variants (blue: *ATM* P1054R and L2307F, and *MPZL2* deletion rs13915729), and ClinVar variants of uncertain significance (black). Gene exons (green blocks), introns (horizontal green lines), untranslated regions (UTRs, orange blocks), and protein domain/motif (framed rectangles) are shown. The length of the gene (kb) and protein (number of amino acids, AA) are shown to the right.

The topmost risk-conferring variant is a missense SNV, p.V2716A, in the phosphatidylinositol 3-kinase (PI3K) catalytic domain of *ATM* (Ataxia telangiectasia mutated; OMIM 607585, UniProt Q13315). This pathogenic variant (rs587782652) is exceedingly rare in the gnomAD, with MAF 0.0021% and 0.0054% in non-cancer controls and NFE population, respectively. In our combined datasets, this variant presented in 0.05% of LCs and 0.003% controls, with remarkably high effect sizes (OR 19.55, 95%CI 5.04–75.6; *P*-value 1.7e-05). LC carriers of this variant were predominately enriched in smokers (8/9 carriers), AD (7/9 carriers), and early-onset (6/9 carriers; mean 55 yrs). Further, four additional rare deleterious variants were observed in *ATM* (Fig. 1 and Supplementary Table 3). No LD is present among these variants and the candidate p.V2716A (Supplementary Table 4).

The second risk variant is c.*28delT in the 3′ UTR of *POMC* (Pro-opiomelanocortin; OMIM 176830, UniProt # P01189). The MAF of this 2 bp del (rs756770132) were 0.086%/0.17% in gnomAD non-cancer/NFE controls; while in our dataset presented in 0.66% of LCs and 0.15% of controls, conferring a 4-fold risk for carriers (OR 4.33, 95%CI 2.03–9.24; *P*-value 0.00015). Although reported as VUS in ClinVar, this 3′ UTR del is located in a critical site computationally predicted to be targets of several miRNAs by the TargetScan^35^, including hsa-miR-149-3p and hsa-mir-625-5p. We also observed four additional rare deleterious variants in the TRICL set (Fig. 1 and Supplementary Table 3).

The third novel risk variant is p.N364M fs*67del in *STAU2* (Staufen homolog 2; OMIM 605920, UniProt Q9NUL3). This del (rs746501298) is very rare in gnomAD (MAF 0.011%/0.0027% in non-cancer/NFE population controls), but presented in 1.02% of LCs and 0.02% of non-cancer controls (OR 4.48, 95%CI 1.73–11.55; *P*-value 0.0019). It was predicted to disrupt the double-stranded RNA-binding motif (DSRM; Fig. 1) which plays a critical role in RNA editing. This del is also reported in the Catalogue of Somatic Mutations In Cancer (COSMIC, # COSM253104).

The fourth and fifth variants are two pathogenic, truncating deletions ─ p.I24M fs*22del (rs752672077) in *MPZL2* (Myelin protein zero-like protein 2, or Epithelial v-like antigen 1 [EVA1]; OMIM 604873, UniProt O60487), and p.Q334V fs*3del (rs563947699) in *MLNR* (Motilin receptor; OMIM 602885, UniProt O43193) ─ with effects sizes of 3.88 (95% CI 1.71–8.8) and 2.69 (95% CI 1.33–5.43), respectively. The *MPZL2* deletion was close to the Immunoglobulin-like antibody Variable domain (Ig-V; Fig. 1) which is involved in thymocyte development^36^. In gnomAD, MAF was the highest in the Ashkenazi Jewish (AJ, 0.38%) than other populations, including NFE (0.123%), Latino (0.028%), and African (0.012%). Additionally, a start-loss p.M1T of *MPZL2* was present in two LCs (Fig. 1 and Supplementary Table 3).

Other interesting candidates from the discovery (Supplementary Table 1), include 1) two VUS ins, *TP63* c.*2550insT (rs772929136) and *CHEK2* c.*2insC (rs749257861), both were located in the 3′ UTR; however, no genotype data/coverage were available in validation sets; 2) a protective effect pathogenic variant, *CHEK2* p.S428F (rs137853011), that was non-significant in the meta-analysis (OR 0.41, 95% CI 0.13–1.31, *P*-value 0.13).

### Candidate gene prioritization

As shown in Table 2, of the 24 candidate genes, the most evolutionarily constrained (intolerance) genes with the lowest LoF observed/expected (o/e) values were *PHF13*, *TP63*, and *STAU2*; whereas the genes with the highest LC-correlated PhoRank scores were *CHEK2*, *ATM*, *TP63*, and *MME*. The most interesting protein interaction network consists of eight genes and is centered on three known DNA damage response genes, *CHEK2-ATM-TP63*, linking five other genes (Supplementary Fig. 5). GO enrichment analysis highlighted genes involved in replicative senescence (which triggers a DNA damage response); whereas KEGG pathway analysis revealed that genes were involved in small cell LC (Supplementary Table 5).

### Endogenous DNA damage assay

**Fig. 2:**
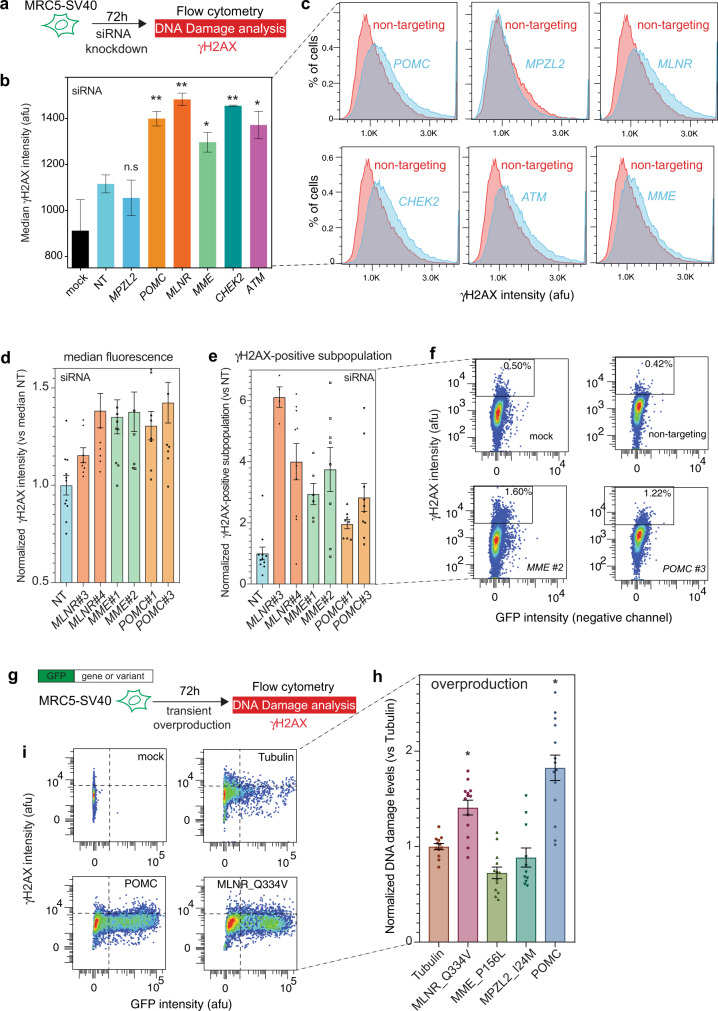
Discovery of DNA damageome genes/proteins and variants. **a** siRNA knockdown endogenous DNA damage assay scheme. **b** Increased DNA damage (γH2AX) levels in five out of the six genes knockdowns (mean ± SEM, *n* = 2~4), *MLNR, CHEK2, POMC, ATM*, and *MME*, compared with non-targeting (NT) siRNA control. There is no increasing DNA damage in *MPZL2* knockdown cells. **c** Representative flow histograms showing higher γH2AX levels in gene knockdowns. **d**–**f** *MLNR*, *MME*, and *POMC* knockdown by two individual siRNAs confirmed the DNA damage-up phenotypes by pooled siRNAs in **b**. DNA damage quantified by **d** median fluorescence intensity or **e** DNA-damage positive subpopulation. **f** Examples of flow cytometry dot plots showing DNA-damage positive subpopulation. **g** Overproduction endogenous DNA damage assay scheme. **h** Wildtype POMC and mutant MLNR p.Q334V fs*3del overproduction promote DNA damage. GFP-Tubulin as a control. **i** Representative histograms of (g). *P-value < 0.05, **P-value < 0.01, n.s not significant *(P*-value > 0.05).

Large conserved networks of *E. coli* and human proteins were recently discovered to promote endogenous DNA damage when overproduced^37^. These networks are known as DNA damageome proteins (DDPs)^37^. The DNA damageome also includes LoF variants that show DNA damage-up phenotypes^38^, most of which are not directly related to DNA repair but rather participate in the DNA damage production. We selected six prioritized genes for the assay: *CHEK2*, *ATM, MPZL2*, *MLNR*, *POMC*, and *MME*. We discovered the knockdown of five genes, overproduction of the mutant *MLNR* p.Q334V fs*3del and wildtype POMC promote DNA damage. Specifically, we first used pooled small interfering RNAs (siRNAs) that minimize off-target effects, and observed significantly increased DNA damage levels (γH2AX) for 5/6 genes (Fig. 2a–c), including two well-known DNA repair genes (*CHEK2* and *ATM)* and three newly discovered DDPs (*POMC*, *MLNR*, and *MME*). By contrast, the knockdown of *MPZL2* did not affect DNA damage. For the three newly discovered DDPs, we further validated their DNA damage phenotypes using different individual siRNAs (Fig. 2d–f). Moreover, overproducing the mutant *MLNR* p.Q334V fs*3del and the wildtype POMC open reading frame (ORF) from the plasmid promote DNA damage in the lung fibroblast-derived cell line (Fig. 2g–i).

## Discussion

Our analyses led to the identification of 25 rare deleterious candidates (in 24 genes) that may be associated with LC susceptibility. Of the five validated variants, we rediscovered two pathogenic variants mapped to known LC susceptibility loci, *ATM* p.V2716A and *MPZL2* p.I24M fs*22del; and identified three deletions in novel LC susceptibility genes, *POMC* 3′ UTR c.*28delT, *STAU2* p.N364M fs*67del, and *MLNR* p.Q334V fs*3del. Our GxE analysis also suggests some of these associations may be further modified by smoking (*MLNR* p.Q334V fs*3del and *MOB3A* p.F69_I75del) and FHLC (*TXNDC15* p.E9G fs*68del). Additionally, our assays of cellular DNA damage identified *POMC* and *MLNR* as part of the DNA damageome, and confirmed a double-strand break repair role of *ATM*.

This study confirms a robust association between LC susceptibility and *ATM* and discovered a new pathogenic p.V2716A, that reside in the PI3K catalytic domain. We also found this association is more evident in AD, which is consistent with several previous studies^21,39,40^. *ATM* is a critical first responder to DNA damage in the cell and essential for genome stability. Several association studies have indicated that common variants of *ATM* are linked to cancer susceptibility, including LC^41–43^. Expression of the PI3K domain in ataxia-telangiectasia cells resulted in complemented radiosensitivity and reduced chromosomal breakage after irradiation^44–46^, suggesting the PI3K domain contains many of the significant activity of *ATM*^47^. Our DNA damage assay also shows elevated DNA damage in lung fibroblasts confirming the previous finding that *ATM* defective cells accumulate more double-strand breaks^48^. Further, the presence of additional rare deleterious variants, together with previously identified p.P1054R^31^ and p.L2307F^21^, strongly suggests that the *ATM* gene plays a role in LC susceptibility.

Another known LC locus we rediscovered is *MPZL2* (also called Epithelial v-like antigen 1, EVA), and the pathogenic frameshift p.I24M fs*22del. *MPZL2* is located at 11q23.3, a known GWAS locus for LC^31,49^ and hearing loss^50,51^. *MPZL2* is one of the top candidate target genes at this locus based on the expression quantitative trait loci (eQTLs) mapping^31^. *MPZL2* is a member of the immunoglobulin superfamily, preferentially expressed in lung and thymus epithelium with a potential role as a favorable prognostic marker in thyroid cancer^52^. Interestingly, the MAF of p.I24M fs*22del in the AJ population was 5-fold higher than the general population in gnomAD. There are several examples where rare causal variants (e.g., variants in the *P53*, *CFTR*, and *BRCA1*/*2*) have higher frequencies within the AJ population^53–56^. In our DNA damage assay, *MPZL2* expression levels do not affect endogenous DNA damage in lung fibroblasts, implying the need to investigate alternative mechanisms in future functional studies.

The most consistent and interesting findings are two new deletions: *POMC* 3′ UTR c.*28delT and *MLNR* p.Q334V fs*3del. *POMC* encodes a polypeptide hormone precursor that regulating energy metabolism, nicotinic-induced weight loss, and immune reactions^57–59^. In particular, *POMC* plays a role in UV-induced DNA damage through interactions with *TP53* and is associated with skin cancer susceptibility^60–64^. Abnormal expression of *POMC* was a poor prognostic marker for LC^65–68^. Using in vitro models, Derghal et al. evaluated putative miRNA (i.e., miR-383, miR-384-3p, and miR-488) and found them physically bind to the 3′ UTR mRNA and regulate *POMC* expression in several neuronal subtypes^69^. Our DNA damage assay showed both downregulation and overproduction of wildtype POMC promotes endogenous DNA damage. Whether and how the c.*28delT affects *POMC* expression and their putative role to LC risk merit further mechanistic investigation. *MLNR* is a member of the G-protein coupled receptor 1 family, and known for regulating gastrointestinal activity^70^. *MLNR* variants and dysregulation have been implicated in lung occult small cell carcinoma, bile duct cancer^71^, and head and neck cancer^72^. Our overproduction results of the *MLNR* p.Q334V fs*3del suggest a dominant-negative role in terms of DNA damage promotion. Collectively, these findings suggesting *POMC* and *MLNR*, while both functions in multiple cellular processes, might also share their various effects on DNA damage.

Although the pathogenic variant, *CHEK2* p.S428F with lower LC risk is not statistically significant in the meta-analysis, its protective effect is consistent with another known pathogenic low-frequency variant, *CHEK2* p.I157T, associated with reduced risk of smoking-related cancers (lung, laryngeal, urinary, and upper aerodigestive tract)^18,73–75^. In contrast, both p.I157T and p.S428F showed an increased risk of breast cancer^75–79^. The mechanism underlying this effect is an ongoing question with unknown impact, perhaps related to smoking exposure and cell cycle checkpoint signaling/apoptosis^75^. STAU2 is a double-stranded RNA-binding protein and a major regulator of mRNA transport, decay, and translation^80^. It was reported that *STAU2* downregulation enhances levels of DNA damage (γH2AX) and promotes apoptosis (PARP1 cleavage) in camptothecin-treated cells^81,82^. The role of *STAU2* in LC requires future investigations.

A main strength of the study is the focus on LC patients with extreme phenotypes of known risk factors (i.e., early-onset, FHLC, or familial cases in high-risk families), which provide >5 times statistical power^10^. Another strength was the relatively large sample size, which is by far the largest collection of LC rare variant analysis to our knowledge. It should be noted however that our study still has limited power to detect association for ultra-rare variants and those candidates (16/25) that could not be assessed in the validation. Third, our exome plus customized captures (50 Mb + 250 kb) in the discovery offers an efficient method for analyzing known susceptibility regions at greater depth and better coverage, particularly for indels that are often poorly captured in GWAS. Last, we have focused on the investigation of predicted LoF variants which provide directionality of effect. Notably, 14/25 candidates we identified were frameshift deletions that result in either truncated proteins or nonsense-mediated mRNA decay. In the discovery, we observed non-coding variants reside in regulatory regions that may influence target gene expression; however, the lack of population frequency information and insufficient coverage in the validation, limits our ability to explore this aspect for some non-coding variants.

There exist various challenges using the gnomAD as controls, including lack of individual-level data, inability to perform GxE interaction, gene-burden tests, and differences in platforms/coverage. Additionally, there were some racial differences in non-white between TCGA cases (27%) and gnomAD controls (30%), that could cause biased effect sizes in the meta-analysis. Genetic ancestry analysis shows 90% TCGA-LCs were inferred as genetic European ancestry^83^. However, it is possible that a small portion of European ancestry TCGA-patients has AJ origin, given that 7% of ovarian cancer^84^ and 24% of endometrial cancer^85^ are of AJ heritage. It is of note that in our dataset, none of the variant allele carriers of the 25 candidates were found to have African-ancestry. Therefore, we expect this potential population stratification effect to be relatively small on rare variant associations, particularly in non-Africans that have not experienced severe population bottlenecks^86–88^.

Although we demonstrated strong joint-effect of the 25 potential candidates (Supplementary Table 2), it is challenging to detect tissue-specific eQTL effects, identify mutational signatures, or construct polygenic risk score (PRS) based on these rare or ultra-rare candidates, due to their low frequencies and weak LD among rare or with common variants. We found some lung-tissue specific eQTL variants from The Genotype-Tissue Expression project (GTEx): three SNPs for *ATM*, 61 SNPs for *POMC*, 75 SNPs for *MPZL2*, and 141 SNPs for *STAU2*; but none of them overlap or are in LD with the 25 candidates we are reporting. Future studies could integrate single-cell transcriptomic sequencing and epigenomic maps in cells and tissues relevant to LC, to establish mutation signatures (i.e., DNA mismatch repair) and explore the application of PRS to clinical care.

In conclusion, our results provide evidence that rare deleterious variants with moderate to large effect sizes, in particular *ATM* p.V2716A*, MPZL2* p.I24M fs*22del, *STAU2* p.N364M fs*67del, *POMC* 3′ UTR c.*28delT, and *MLNR* p.Q334V fs*3del, contribute to LC susceptibility. Additional targeted studies using CRISPR/Cas9 mutagenesis could be performed for each variant, to evaluate more comprehensively what its effects are on gene functions and the underlying molecular mechanisms. Future extremely large-scale multi-ancestry studies may also provide additional opportunities to assess ancestry-specific predisposing variants, and discover new genetic alterations with relatively large attributable risk for LC.

## Methods

### Study population in the discovery set

The discovery set included 1094 LC cases and 933 controls from the TRICL study^89^. All study subjects and biospecimens were collected with informed consent under institutional review board (IRB) approved protocols. Subjects were selected from four sites: Harvard School of Public Health (HSPH), International Agency for Research on Cancer (IARC), University of Liverpool, and Mount Sinai Hospital and Princess Margaret Hospital (MSH-PMH) in Toronto^89^. Cases were selected because they reported FHLC (first-degree) or were early-onset (<60 yrs) or had specimens available (Table 1). Never smokers were defined as persons who had smoked fewer than 100 cigarettes in their lifetimes. The ethnicities were inferred using FastPop^90^.

### WES and variant calling in the discovery set

WES was performed using captures with Agilent SureSelect v5 (50 Mb, Agilent Technologies) and custom capture targeted known LC-GWAS region^91,92^ (250 kb). Germline DNA was sequenced at the Center for Inherited Disease Research. The mean on-target coverage was 52x for each sequencing experiment and greater than 97% of on-target bases had a depth greater than 10x. Sequence reads were mapped to the human reference GRCh37/hg19 using the Burrows-Wheeler Aligner. SNVs and indels were called based on the union of raw GATK v3.3-0 and Atlas2. QC process involved the following user-definable criteria: i) low-complexity repeats and segmental duplications were filtered out; ii) quality score ≥20, depth ≥10, and AB ≥ 0.2 for heterozygous calls; iii) call rate ≥0.85; and iv) samples with abnormal heterozygosity rate, sex discordance, <95% completion rates, and unexpected relatedness (identity-by-state >10%) were filtered out.

### Rare variant filtering and functional annotation in the discovery set

Following variant calling, rare variants were further enriched by the application of three-steps: i) Variant with MAF < 1% in the gnomAD (NFE ancestry, v2.1); ii) Variants class, including missense, protein-truncating, and regulatory; and iii) Mutation effects, i.e., variant results in protein truncation and predicted to be deleterious from 4/6 prediction tools (SIFT, Polyphen-2, MutationTaster, MutationAssessor, FATHMM, and FATHMM-MKL). The miRNAs putatively bound to the sequence containing UTR variants were identified by the TargetScan^35^. We additionally incorporated rare variants classified as pathogenic, likely pathogenic, or VUS from the ClinVar database, which compiles clinically observed human variants.

### Single variant association test in the discovery set

For variants derived from the above automated filtering schema, we conducted the association test using Fisher’s exact test. We used the Genome Browser (Golden Helix) visualization tool to verify the presence of the potential candidates in each carrier. By manual review of the variants’ coverage plot (read depth) and pile-up plot (read alignment), we rule out low-confidence variants resulting from mapping error, strand bias, and weak exon conservation.

### Gene–environment interaction and gene-based burden analysis in the discovery set

For the candidates identified from the association test, we performed G×E interaction (i.e., age-onset, sex, smoking status, pack-years, and FHLC), using the mixed linear regression model. To measure the cumulative effect of the rare deleterious variants within the gene, we performed collapsing tests using the CMC and the KBAC tests^93,94^.

### Study populations in the validation sets and meta-analysis

The candidate variants were further examined in seven validation datasets, aggregated from different centers and across several platforms (four WES data and three genome-wide genotyping datasets as shown in Table 1). We tabulated the variant carrier counts per candidate and performed meta-analyses using the inverse-variance-weighted fixed-effects (assume the true effect size is the same in all studies).

1. **GELCC** study (Genetic Epidemiology of LC Consortium, 380 LCs): This included 122 familial and 258 sporadic LC cases. i) Familial LC Study Subjects (dbGaP phs000629.v1.p1). The familial cases were selected from high-risk LC families with at least two first-degree relatives affected with LC^95^. The GELCC study population and recruitment scheme have been described in detail previously^96^. Samples and data were collected by the familial LC recruitment sites of the GELCC, that included the University of Cincinnati, University of Colorado Health Science Center, Karmanos Cancer Institute at Wayne State University, Louisiana State University Health Sciences Center-New Orleans, Mayo Clinic, University of Toledo, Johns Hopkins University, and Saccomanno Research Institute. ii) Sporadic LC Study Subjects. The sporadic LC patients were selected from our previous WES study^19,20^, including samples from the HSPH, Baylor College of Medicine (BCM), and MD Anderson Cancer Center (MDACC). Germline DNA was sequenced utilizing NimbleGen VCRome 2.1 (Roche)^19,20^, and HumanOmniExpressExome (Illumina)^95^.
2. **TCGA** (The Cancer Genome Atlas cohort, 1015 LCs): this public germline WES dataset includes non-tumor DNA from 577 AD and 438 SCC (dbGaP Phs000178.v9.p8), using Agilent SureSelect (Agilent Technologies) and NimbleGen SeqCap (Roche).
3. **COPDGene** (Genetic Epidemiology of COPD Study^97^, 318 controls): controls were selected to be white, smokers with normal lung function data (defined as post-bronchodilator Forced Expiratory Volume in 1 s [FEV_1_] ≥ 0 80% predicted, FEV1/FVC ≥ 0.7), and with smoking histories ≥10 pack-years; WES utilized NimbleGen VCRome 2.1 (Roche)^19,20^.
4. **GnomAD** (the Genome Aggregation Database, 134,187 controls): we restricted our analyses to non-cancer individuals (excluded individuals from cancer cohort studies, such as the TCGA cohort), resulting in a data subset of 118,479 exomes and 15,708 whole genomes; multiple exome captures were utilized including Nimblegen SeqCap (Roche), Agilent SureSelect (Agilent Technologies), and Illumina Exome BeadChip (Illumina).
5. **Oncoarray** case–control study (17,878 LCs vs. 13,425 controls; dbGaP phs001273): The OncoArray consortium is a network created to increase understanding of the genetic architecture of common cancers. We restricted our analyses to European descent subjects (Supplementary Fig. 1)^98–100^; participants were obtained from 29 LC studies across North America and Europe, and genotyped on OncoArray-500K BeadChip (Illumina). There were 1162 participants in the OncoArray consortium who were also exome-sequenced in the TRICL discovery, and therefore these samples were excluded from the analysis in the validation phase.
6. **Affymetrix** case–control studies (5364 LCs vs. 5724 controls; dbGaP phs001681.v1.p1). This is a large pooled sample was assembled consisting of 10 independent case–control studies which previously described elsewhere^99,101^. Study participants were genotyped on an Axiom Exome Plus Array (Affymetrix)^99,101^, which contains a custom panel of key LC GWAS markers, and rare coding SNVs and indels^102^. There were 992 participants in the Affymetrix that were also exome-sequenced in the TRICL discovery, and therefore these samples were excluded from the analysis in the validation phase.
7. **UKB** (UK Biobank cohort^103^; 2166 LCs vs. 401,453 controls): we restricted our analyses to non-cancer controls and LC cases; individuals were genotyped on UK BiLEVE Axiom Array and UK Biobank Axiom Array (Affymetrix)^103,104^.

### Gene prioritization based on functional annotations and protein interactions network

To better reprioritize genes and candidates, we used three prioritization tools: 1) Gene evolutionary constraint to LoF variation, which using the o/e ratio from the gnomAD. 2) Phevor PhoRank algorithm^105^, which ranks the genes based on their phenotypic relevance as defined by diverse biomedical ontologies. 3) Protein–Protein interactions (PPI) network using the STRING database^106^, with an interaction score cut-off ≥0.15 (low confidence).

### Functional evaluation of candidate genes using endogenous DNA damage assay

Endogenous DNA damage is proposed to drive cancers by genome instability — a hallmark of cancer^37,38^. To test whether knockdown or overexpression of the candidate genes or variants induces endogenous DNA damage, we performed flow cytometric assays to measure γH2AX levels, a DNA double-strand-break marker^107^, following siRNA knockdown and overproduction of GFP fusions of proteins of interest.

1. **Human cell lines and reagents**. MRC5-SV40, a human lung fibroblasts derived cell line was maintained in standard Dulbecco’s modified Eagle’s medium with 10% fetal bovine serum, 2 mM L-glutamine, 100 μg/mL penicillin, and 100 μg/mL streptomycin^37,38^. The cell line was authenticated by ATCC STR analysis and routinely check to be mycoplasma-free. MLNR p.Q334V fs*3del, MME p.P156L fs, MPZL2 p.I24M fs*22del, and full-length wildtype POMC entry clones for gateway cloning was synthesized, sequence-verified, and cloned into pDONR223 (Invitrogen) by Genscipt. All the above clones were further subcloned into an N-terminal GFP tagged vector (pcDNA6.2/N-EmGFP-DEST, Invitrogen), using Gateway LR Clonase II Enzyme Mix (Invitrogen). Overexpression plasmids transfections were performed using GenJet In Vitro DNA Transfection Reagent Ver. II (# SL100489, SignaGen). Non-targeting pool siRNA (D-001810-10), SMARTpool siRNAs each containing four targeting sequences of *MME, MLNR, POMC, ATM, CHEK2, and MPZL2*, sets of 4 siRNAs targeting *MME, MLNR*, and *POMC* were purchased from Dharmacon. The target sequences for *MME, MLNR*, and *POMC* are as follows: #1 MME (GGAGGCUGGUUGAAACGUA), #2 MME (GAACCUAUAGGCCAGAGUA), #3 MME (AAAGAUGAGUGGAUAAGUG), #4 MME (GACAGCACCUUAAUGGAAU); #1 MLNR (GCGCUAACGUGAAGACGAU), #2 MLNR (GCGCAUCUAUCAACCCAAU), #3 MLNR (CAUCGUCGCUCUGCAACUU), #4 MLNR (GAAGAUUCGCGGAUGAUGU); #1 POMC (GACAAGCGCUACGGCGGUU), #2 POMC (CAGUGAAGGUGUACCCUAA), #3 POMC (GGCCGAGACUCCCAUGUUC), #4 POMC (CUACAAGAAGGGCGAGUGA). siRNA transfections were carried out with lipofectamine RNAiMax Transfection Reagent (#13778075, Invitrogen), following the manufacturer’s recommendations. SMARTpool ON-TARGET*plus* siRNA was designed and modified for greater specificity and reduce off-targets up to 90% utilizing a dual-strand modification.
2. **Real-time quantitative reverse transcription PCR (RT-qPCR)**. Knockdown efficiency was quantified by RT-qPCR and shown in Supplementary Fig. 6. RNeasy mini kit (Qiagen #74106) was used to extract total RNA from cells 72 h post siRNA transfection or protein overproduction. 300 ng of total RNA from each sample was used to synthesize cDNA by the Superscript III first-strand synthesis system (Invitrogen, #18080051). The qPCR reactions were performed using iTaq Universal SYBR Green Supermix (BioRad #172-5121) on a QuantStudio 3 Real-Time PCR System (Applied Biosystems). For each gene, three replicates were analyzed and the average threshold cycle (Ct) was calculated. The relative expression levels were calculated with the 2–ΔΔCt method^108^. Primers used included *GAPDH* (housekeeping gene) forward: CAA TGA CCC CTT CAT TGA CC; *GAPDH* reverse: GAT CTC GCT CCT GGA AGA TG; *POMC* forward: GCC AGT GTC AGG ACC TCA C; *POMC* reverse: GGG AAC ATG GGA GTC TCG G; *CHEK2* forward: TCT CGG GAG TCG GAT GTT GAG; *CHEK2* reverse: CCT GAG TGG ACA CTG TCT CTA A; *ATM* forward: GGC TAT TCA GTG TGC GAG ACA; *ATM* reverse: TGG CTC CTT TCG GAT GAT GGA; *MPZL2 f*orward: TTA ATG GGA CAG ATG CTC GGT; *MPZL2* reverse: AAG ACA CCC GGT CCT TAA ACC; *MME* forward: AGA AGA AAC AGC GAT GGA CTC C; *MME* reverse: CAT AGA GTG CGA TCA TTG TCA CA; *MLNR* forward (siRNA): CTG AGC GCA TCT ATC AAC CCA; *MLNR* reverse (siRNA): TCC CAT CGT CTT CAC GTT AGC; *MLNR* forward (overexpression): GTG GTG ACC GTG ATG CTG AT; *MLNR* reverse (overexpression): AGC AGG ATG AGT AGG TCG GA.
3. **Flow-cytometric DNA damage assays**. Sensitive DNA damage assays by flow cytometry were performed as previously described^37,38^. γH2AX primary antibody (Sigma, Catalog #05-636) and goat anti-mouse secondary antibody, Alexa Fluor 647 (Thermo Fisher, Catalog #A21236) were used to stain cells. Stained cells were then analyzed by a BD LSRFortessa flow cytometer. FCS files were analyzed by FlowJo 10.5 software. For siRNA experiments, cells were collected 72 h post transfection and median fluorescence intensity was quantified. Also, to quantify the DNA-damage positive subpopulations, 0.5% of the mock cells were gated as the γH2AX threshold as previously demonstrated. The percentage of γH2AX positive cells in each sample was calculated and compared to its corresponding non-targeting siRNA control. For overproduction experiments, mock-transfected cells were used to set the gates to determine the GFP and γH2AX positive cells. 0.5% of the mock cells were gated as the γH2AX threshold. The DNA-damage ratios by protein overproduction for 72 h are calculated as described. Briefly, the damage ratio is defined as (Q2/Q3)/(Q1/Q4), where Q2 is the portion of transfected γH2AX-positive cells; Q3 is the portion of transfected, γH2AX -negative cells; Q1 is the portion of untransfected, γH2AX-positive cells; and Q4 is the portion of untransfected, γH2AX-negative cells. The DNA damage ratios by candidate protein overproduction were compared with GFP-Tubulin as previously described.

### Reporting summary

Further information on research design is available in the Nature Research Reporting Summary linked to this article.

## Supplementary information

Reporting Summary

Supplementary Figures and Tables

## Data Availability

The data generated and/or analyzed during the related study are described in the figshare metadata record: 10.6084/m9.figshare.13280387^109^. The data that support the findings of this study are available via the dbGaP (database of genotypes and phenotypes) repository. The data are controlled-access, so interested parties will need to request access — information on how to do so can be found on pages linked to below. The access numbers are https://identifiers.org/dbgap:phs000878.v2.p1^110^ for Transdisciplinary Research in Cancer of the Lung (TRICL) study, https://identifiers.org/dbgap:phs001273.v1.p1^111^ for the OncoArray study, https://identifiers.org/dbgap:phs001681.v1.p1^112^ for the Affymetrix study, https://identifiers.org/dbgap:phs000629.v1.p1^113^ for part of the Genetic Epidemiology of Lung Cancer Consortium (GELCC) study, and https://identifiers.org/dbgap:phs000178.v9.p8^114^ for The Cancer Genome Atlas (TCGA) study. Two files are not publicly available in order to protect patient privacy. These are: ‘TRICL WES.xlsx’ (underlying Supplementary Table 2 and Supplementary Fig. 3) and ‘TRICL WES.bam’ (underlying Supplementary Fig. 2). These data are only available to authorized researchers who have submitted an IRB application. Please email the corresponding author for access. Data underlying Supplementary Table 5 and Supplementary Fig. 5 are a publicly available resource available from the STRING (Search Tool for the Retrieval of Interacting Genes) website: http://string-db.org/. The file used in this study was ‘Protein-Protein Interaction Networks Functional Enrichment Analysis-STRING.txt’. Sources of other datasets used in this study are: the UKB dataset is accessible to approved researchers and applications through ukbgene at www.ukbiobank.ac.uk. The GnomAD dataset can be downloaded from the Genome Aggregation Database at https://gnomad.broadinstitute.org/.
