# Supplementary material for: Rare deleterious germline variants and risk of lung cancer
Source: NPJ Precis Oncol. 2021 Feb 16;5:12. doi: 10.1038/s41698-021-00146-7 (PMC7887261; doi:10.1038/s41698-021-00146-7)
Supplement: Supplementary file 1 — Reporting Summary [file 41698_2021_146_MOESM1_ESM.pdf]

nature research

Corresponding author(s): Christopher I. Amos

Last updated by author(s): 09/08/2020

Reporting Summary

Nature Research wishes to improve the reproducibility of the work that we publish. This form provides structure for consistency and transparency in reporting. For further information on Nature Research policies, see our Editorial Policies and the Editorial Policy Checklist.

Statistics

For all statistical analyses, confirm that the following items are present in the figure legend, table legend, main text, or Methods section.

n/a	Confirmed	
☐	☒	The exact sample size (n) for each experimental group/condition, given as a discrete number and unit of measurement
☐	☒	A statement on whether measurements were taken from distinct samples or whether the same sample was measured repeatedly
☐	☒	The statistical test(s) used AND whether they are one- or two-sided<br>*Only common tests should be described solely by name; describe more complex techniques in the Methods section.*
☐	☒	A description of all covariates tested
☐	☒	A description of any assumptions or corrections, such as tests of normality and adjustment for multiple comparisons
☐	☒	A full description of the statistical parameters including central tendency (e.g. means) or other basic estimates (e.g. regression coefficient) AND variation (e.g. standard deviation) or associated estimates of uncertainty (e.g. confidence intervals)
☐	☒	For null hypothesis testing, the test statistic (e.g. F, t, r) with confidence intervals, effect sizes, degrees of freedom and P value noted<br>*Give P values as exact values whenever suitable.*
☐	☒	For Bayesian analysis, information on the choice of priors and Markov chain Monte Carlo settings
☐	☒	For hierarchical and complex designs, identification of the appropriate level for tests and full reporting of outcomes
☐	☒	Estimates of effect sizes (e.g. Cohen's d, Pearson's r), indicating how they were calculated

Our web collection on statistics for biologists contains articles on many of the points above.

Software and code

Policy information about availability of computer code

Data collection	The data that support the findings of this study are available.
Data analysis	Described in Methods, Results, and Supplemental Methods. No custom algorithms or software were used in this study.

For manuscripts utilizing custom algorithms or software that are central to the research but not yet described in published literature, software must be made available to editors and reviewers. We strongly encourage code deposition in a community repository (e.g. GitHub). See the Nature Research guidelines for submitting code & software for further information.

Data

Policy information about availability of data

All manuscripts must include a data availability statement. This statement should provide the following information, where applicable:

- Accession codes, unique identifiers, or web links for publicly available datasets
- A list of figures that have associated raw data
- A description of any restrictions on data availability

The data that support the findings of this study are available. The access numbers are “phs000878.v2.p1” for Transdisciplinary Research in Cancer of the Lung (TRICL) study, “phs001273.v1.p1” for the OncoArray study, “phs001681.v1.p1” for the Affymetrix study, “phs000629.v1.p1” for part of the Genetic Epidemiology of Lung Cancer Consortium (GELCC) study, and “phs000178.v9.p8” for The Cancer Genome Atlas (TCGA) study.

Field-specific reporting

Please select the one below that is the best fit for your research. If you are not sure, read the appropriate sections before making your selection.

☒ Life sciences ☐ Behavioural & social sciences ☐ Ecological, evolutionary & environmental sciences

For a reference copy of the document with all sections, see nature.com/documents/nr-reporting-summary-flat.pdf

Life sciences study design

All studies must disclose on these points even when the disclosure is negative.

Sample size	Study populations in discovery set (1,045 lung cancer cases and 885 controls) and validation sets (26,803 lung cancer cases and 555,107 controls)
Data exclusions	Data exclusions mentioned in the Methods and Supplemental Methods
Replication	Replicates mentioned in the Supplemental Methods and Tables
Randomization	N/A
Blinding	Sequencing and genotype experiments were performed blinded.

Reporting for specific materials, systems and methods

We require information from authors about some types of materials, experimental systems and methods used in many studies. Here, indicate whether each material, system or method listed is relevant to your study. If you are not sure if a list item applies to your research, read the appropriate section before selecting a response.

Materials & experimental systems

n/a	Involved in the study	
☐	☒	Antibodies
☐	☒	Eukaryotic cell lines
☒	☐	Palaeontology and archaeology
☒	☐	Animals and other organisms
☐	☒	Human research participants
☒	☐	Clinical data
☒	☐	Dual use research of concern

Methods

n/a	Involved in the study	
☒	☐	ChIP-seq
☐	☒	Flow cytometry
☒	☐	MRI-based neuroimaging

Antibodies

Antibodies used	Anti-phospho-Histone H2A.X (Ser139) Antibody, clone JBW301 (Sigma, Catalog #05-636) and goat anti-mouse secondary antibody, Alexa Fluor 647 (Thermo Fisher, Catalog #A21236)
Validation	Anti-phospho-Histone H2A.X (Ser139), clone JBW301 is a well published Mouse Monoclonal Antibody validated in ChIP, ICC, IF, WB. This purified mAb is highly specific for phospho-Histone H2A.X (Ser139) also known as H2AXS139p. More than 2000 citations validating this antibody.

Eukaryotic cell lines

Policy information about cell lines

Cell line source(s)	MRC5-SV40 from Steve Jackson Laboratory
Authentication	ATCC STR analysis
Mycoplasma contamination	the cell line used was tested negative for mycoplasma contamination
Commonly misidentified lines (See ICLAC register)	N/A

Human research participants

Policy information about studies involving human research participants

Population characteristics	Described in Methods, Results, Table and Supplemental Methods
Recruitment	Described in Methods and Supplemental Methods
Ethics oversight	All studies were reviewed and approved by institutional ethics review committees at the involved institutions

Note that full information on the approval of the study protocol must also be provided in the manuscript.

Flow Cytometry

Plots

Confirm that:

☐ The axis labels state the marker and fluorochrome used (e.g. CD4-FITC).

☐ The axis scales are clearly visible. Include numbers along axes only for bottom left plot of group (a 'group' is an analysis of identical markers).

☐ All plots are contour plots with outliers or pseudocolor plots.

☒ A numerical value for number of cells or percentage (with statistics) is provided.

Methodology

Sample preparation	Described in the Supplemental Methods under Functional evaluation of candidate genes using endogenous DNA damage assay
Instrument	BD LSRFortessa
Software	Flowjo 10.5
Cell population abundance	No cell sorting was involved
Gating strategy	Described in the Supplemental Methods and illustrated in the Figure 2

☐ Tick this box to confirm that a figure exemplifying the gating strategy is provided in the Supplementary Information.
