# Supplementary material for: Rare deleterious germline variants and risk of lung cancer
Source: NPJ Precis Oncol. 2021 Feb 16;5:12. doi: 10.1038/s41698-021-00146-7 (PMC7887261; doi:10.1038/s41698-021-00146-7)
Supplement: Supplementary file 2 — Supplementary Figures and Tables [file 41698_2021_146_MOESM2_ESM.pdf]

Supplementary Figure 1. Principal component analysis of study subjects

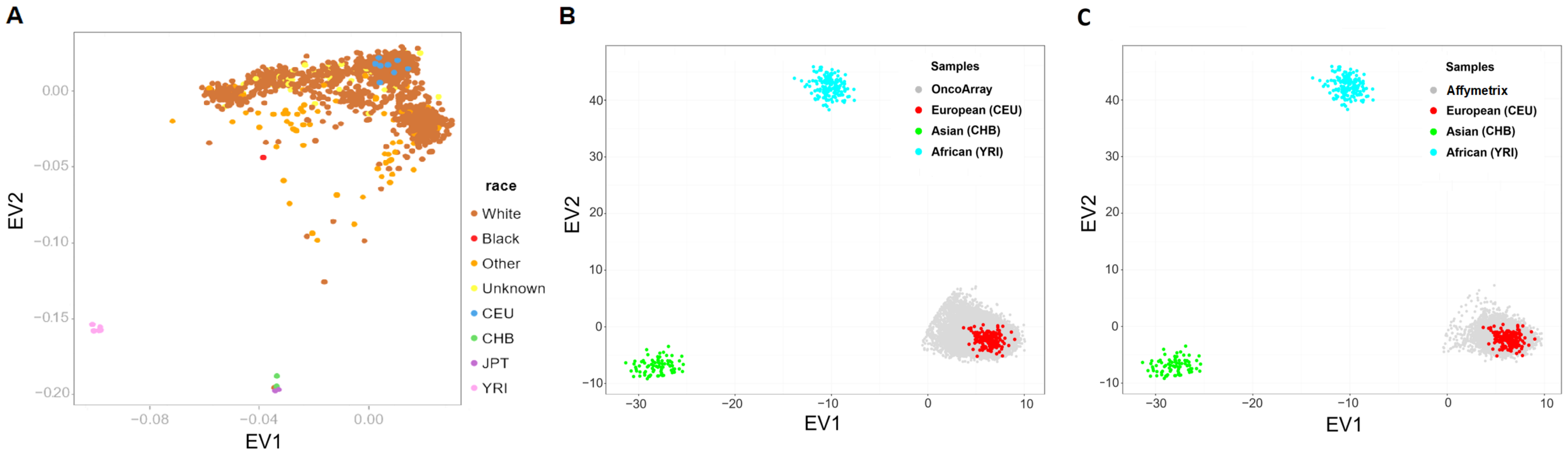

Figure Legend

(A) TRICL study subjects along with 17 HapMap controls: PCA performed on 28,072 high quality variants (LD-pruned, $r^2 < 0.1$), autosomal, bi-allelic SNVs with allele frequency > 5% and call rate > 99% to determine the ancestry of the samples. One self-identified “Black” subject is halfway between the European and African cluster. The majority of “Other” subjects lie in the European-ancestry cluster, some “Other” subjects are between the European and Asian clusters. (**B)** Oncoarray and **(C)** Affymetrix study subjects along with 407 HapMap controls. The analyses for Oncoarray and Affymetrix were restricted to the European population only.

Supplementary Figure 2. Variant map for the top five candidate rare deleterious variants

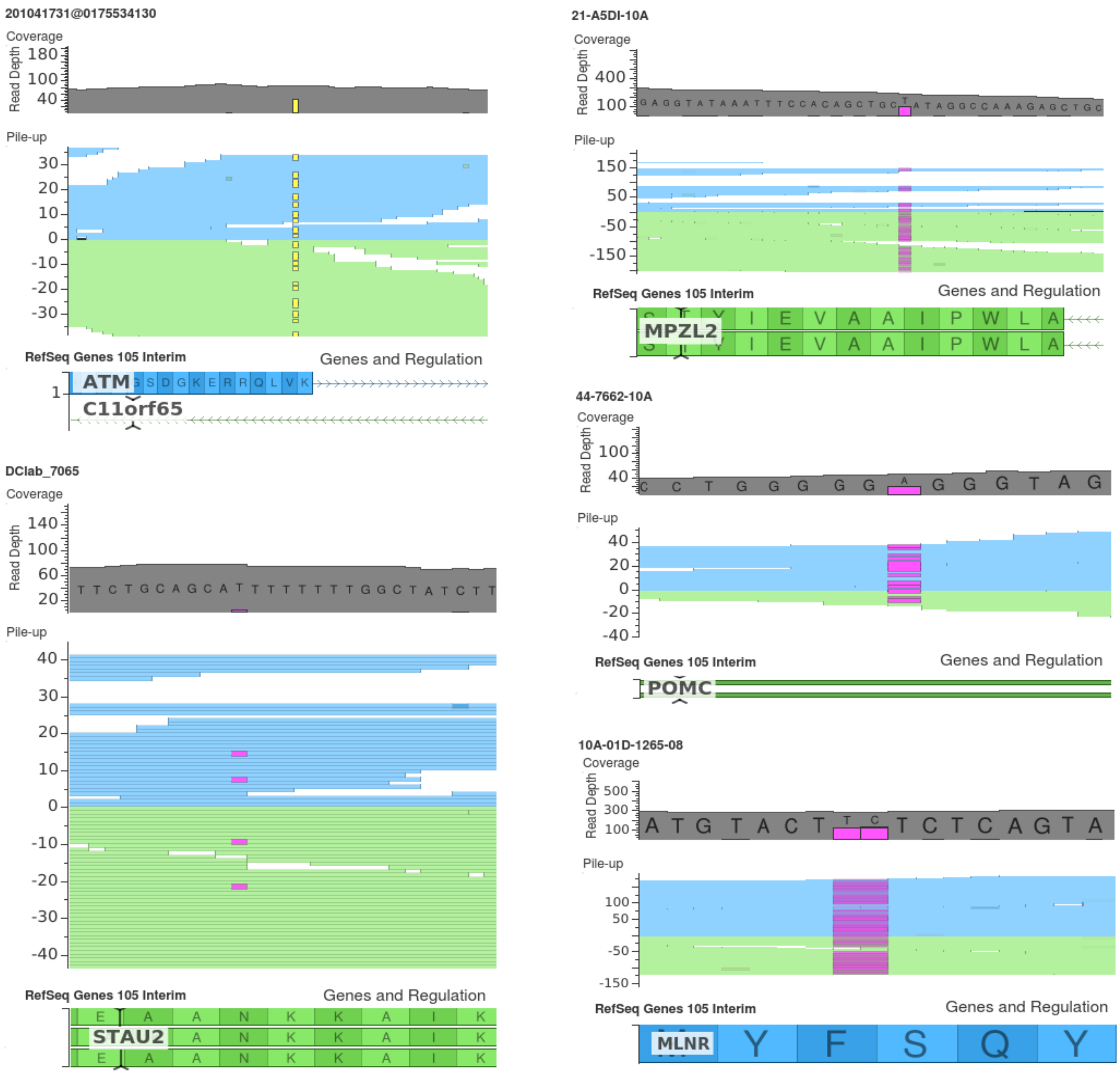

Figure Legend

Variant map visualization was performed for the samples' Binary Sequence Alignment Map (BAM) files using the Genome Browser. *ATM* SNV p.V2716A, *MPZL2* p.I24M frameshift (fs)*22 deletion (del), *POMC* 3' UTR c.*28delT, *STAU2* p.N364M fs*67 del, and *MLNR* p.Q334V fs*3 del. The non-synonymous substitutions (yellow) and deletions (fuchsia) are shown in pile-up (read depth) histograms, which are split into two groups to emphasize the forward (blue) or reverse (green) strand. The gene annotation also shows forward (blue) or reverse (green).

Supplementary Figure 3. Test of correlation between the presence of candidate variants and age

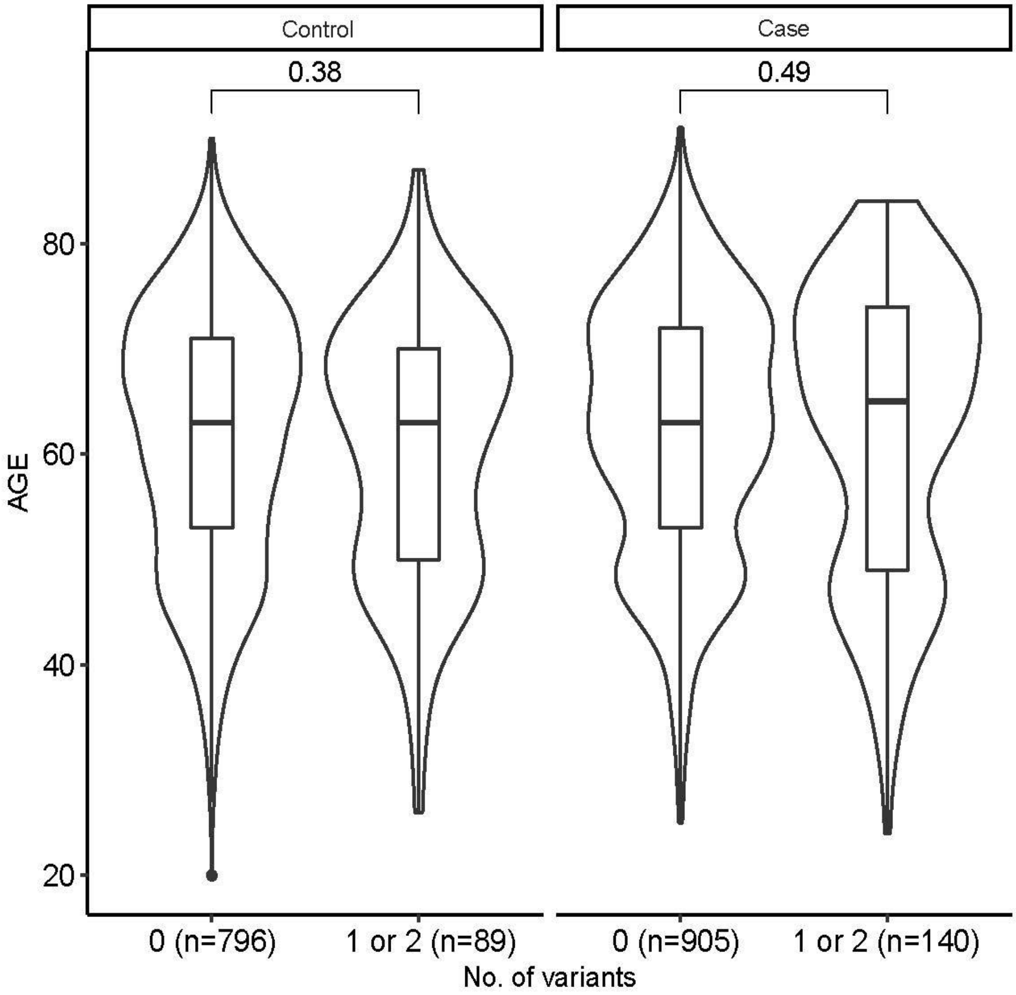

Figure Legend

Age distribution of study subjects carries 0, 1, and 2 variant alleles of the 25 candidates in the TRICL discovery set. Overall, age did not differ significantly between carriers (1 or 2 risk or protective variant alleles) and non-carriers (0 variant alleles). Significances were tested by Wilcoxon rank-sum tests.

Supplementary Figure 4. Gene-based association test in the TRICL discovery set

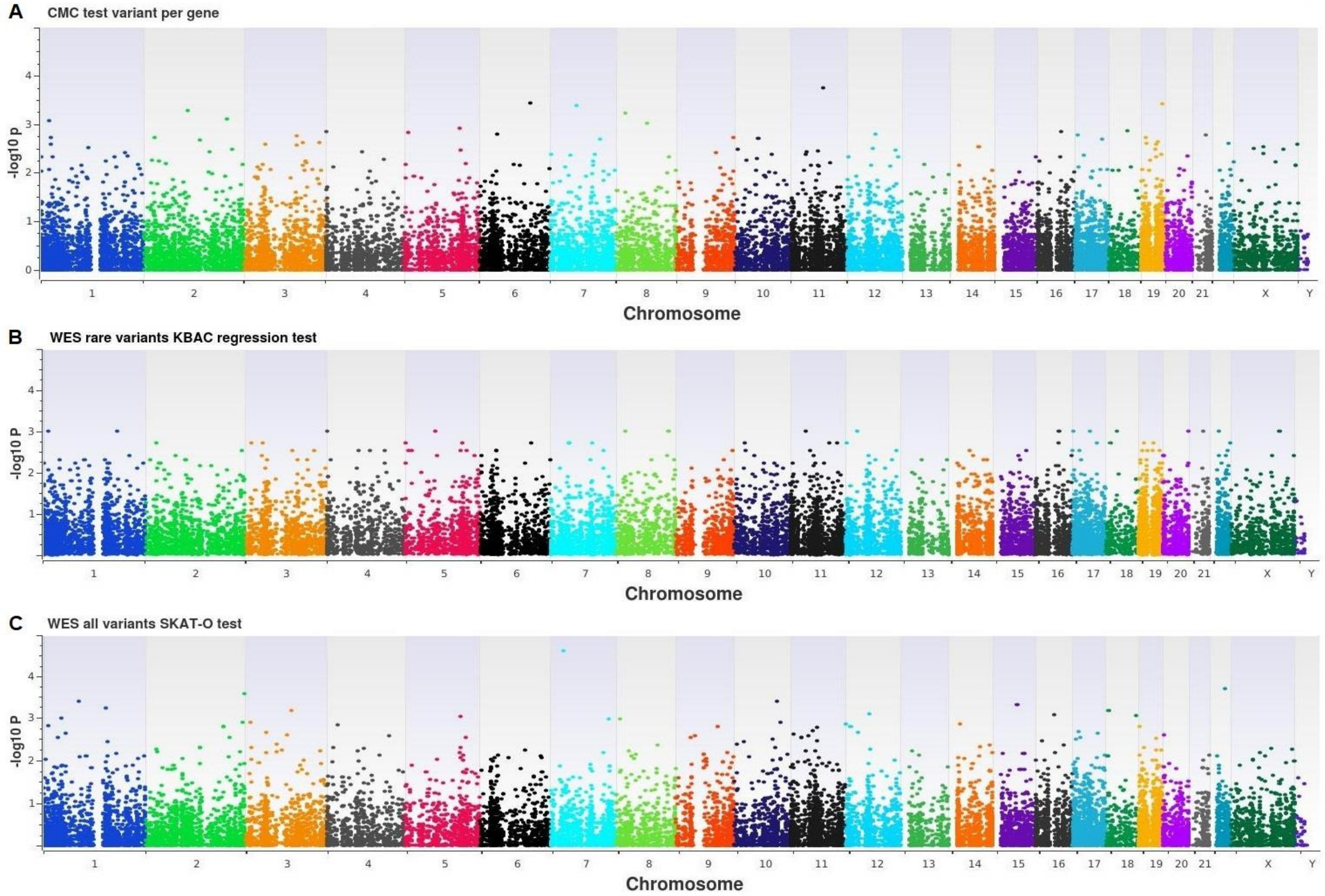

Figure Legend

(A) The Combined Multivariate and Collapsing (CMC) test first bins the rare variants according to the MAF criterion (rare: 0.1 - 1%, very rare: 0.01 - 0.1%, and extremely rare < 0.01%), then collapses each bin and performs multivariate Hotelling’s T2 test. **(B)** The Kernel-Based Adaptive Cluster (KBAC) test first counts multi-marker genotypes of the rare variants within the gene and then performs case-control tests based on the weighted sum of these allele counts. (**C)** The Optimized Sequence Kernel Association Test (SKAT-O) included both common and rare variants within the gene, which weight rare variants more and common variants less. We removed variants that were in high LD (D’ ≥ 0.9; $r^2 \geq 0.7$) with at least one other variant.

Supplementary Figure 5. Protein-protein interaction (PPI) network of candidate genes

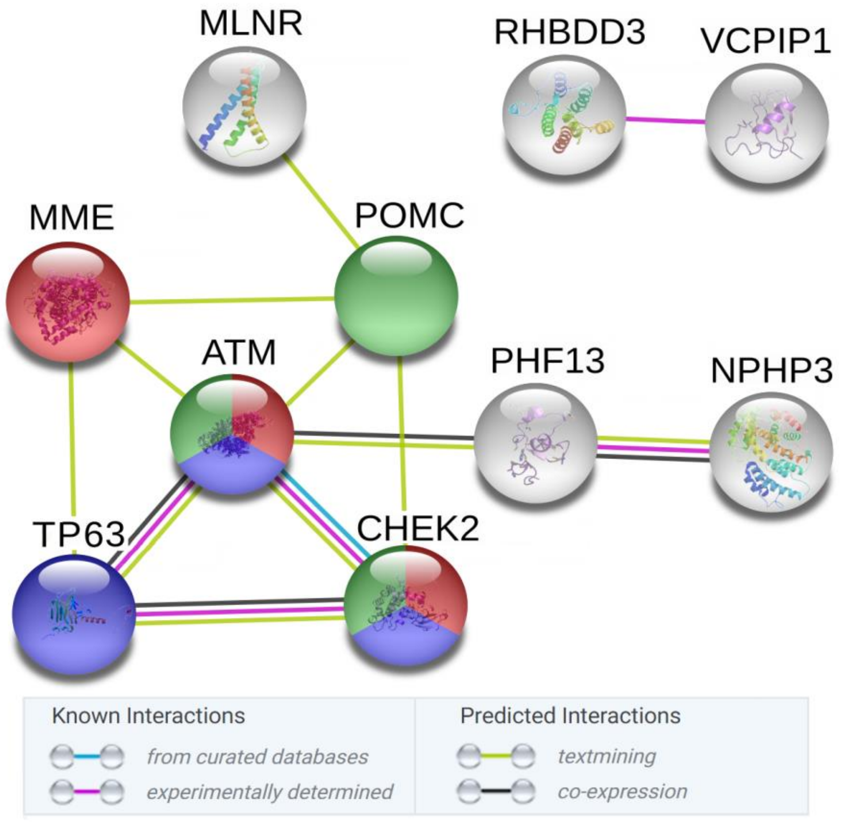

Figure Legend

The PPI (protein-protein interaction) network via STRING (Search Tool for the Retrieval of InteractingGenes) with interaction score cutoff 0.15 (low confidence). The interaction network consists of eight genes and centered on known interactions (experimentally determined, high confidence score > 0.7) among the three DNA damage response genes (blue nodes), *CHEK2 – ATM – TP63*. STRING predicted new suggesting interactions (computational predicted, low confidence score > 0.15) including three genes response to DNA double-strand breaks (green nodes), *POMC – ATM – CHEK2*; and three replicative senescence genes (red nodes), *MME – ATM – CHEK2*. The detailed pair-wise interaction score was listed in **Supplemental Table 5.**

Supplementary Figure 6. Confirmation of decreased or increased mRNA levels of target genes by siRNAs knockdown or overproduction

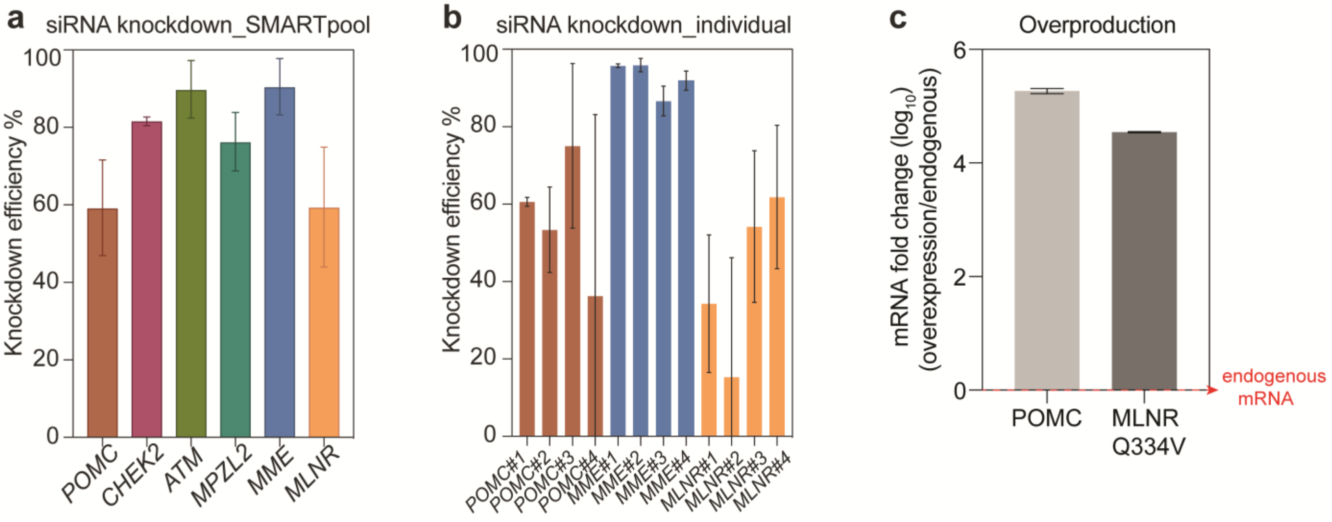

Figure Legend

(A) SMARTpool siRNAs (containing four sequences) targeting six candidate genes; (**B**) Individual four siRNAs targeting *POMC*, *MME*, and *MLNR*. The *POMC* #1,3, *MME* #1, 2, and *MLNR* #3, 4 were chosen for follow-up DNA damage assays in **Figure 2**. siRNA sequences information were described in the **Supplemental Methods**. (**C**) mRNA overexpression in overproduced GFP fusions of wildtype POMC and MLNR p.Q334V fs*3del cells. Red arrow: mRNA levels of *POMC* and *MLNR* from the endogenous locus.

Supplementary Table 1. List of the 75 rare and deleterious variants identified in the TRICL study

**Gene ***	**Marker**		**N. carriers**	**MAF% in GnomAD**		**Association [G x E]**	**Allele**	**Quality**	**Reads**	**Pile-up**
**region**	**GRCh37 / hg19**	**Function effect †, ClinVar annotation**	**LC # / Control**	**non-cancer**	**NFE**	**FDR *P*-value**	**Ref. / Alt.**	**score**	**Alt. / Ref.**	**note**
**25 Candidates**										
PHF13	1:6680069-Del	p.Lys120_Lys121del, In frame	0 / 7	0.0073	0.0079	0.00075	GAA / -	307	5 / 50	High-confidence
BMP8A	1:39991410-Del	p.Asn385del, In frame del	10 / 1	0.165	0.247	0.0074	CAA / -	5827	28 / 46	High-confidence
CASQ2	1:116243913-Del	p.Asp383del In frame, VUS 5x	2 / 11	0.028	0.0304	0.0036	ATC / -	1359	16 / 30	High-confidence
F13B	1:197009871-Del	Splice c.1739del, VUS 1x	6 / 0	0.16	0.1599	0.0066	AAC / -	1387	11 / 25	High-confidence
POMC	**2:25383922-Del**	**c.*28delT, 3' UTR, miRNAs targets, VUS 2x**	6 [4 SC] / 0	**0.086**	**0.17**	0.0066	A / -	3421	20 / 45	High-confidence
NPHP3	3:132408108-Del	Splice acceptor, LoF, Pathogenic 10x	2 / 0	0.028	0.0302	0.105	CT / -	2939	46 / 116	High-confidence
MME/CD10	3:154834479-Del	p.Pro156Leufs*14, LoF fs, Pathogenic 1x	3 [2 AD] / 0	0.024	0.032	0.055	C / -	2558	36 / 56	High-confidence
*TP63*LC*	3:189614842-**Ins**	3' UTR c.*2550ins, VUS 3x	5 [4 AD] / 1	0.29	0.132	0.145	- / T	1950	25 / 36	High-confidence
ENAM	4:71509906-Del	p.Asp921Glufs*32, LoF fs, Pathogenic 1x	3 / 0	0.021	0.0434	0.055	T / -	3860	36 / 125	High-confidence
NKX6A	4:85418968-Ins	p.Ala138_Ala139, In frame Ins	10 / 1	0.109	0.181	0.0075	- / GGCAGAGG CGGAGGA	2505	6 / 32	High-confidence
*TXNDC15 *LC*	5:134211056-Del	p.Glu9Glyfs*68, LoF fs	7 / 22	0.623	0.8577	0.0012 [x FHLC]	A / -	28946	34 / 45	High-confidence
STAU2	**8:74507471-Del**	**p.Asn364Metfs*67, LoF fs**	21 [11 AD] / 4	**0.011**	**0.0027**	0.0015	T / -	191	5 / 40	High-confidence
OR51J1	11:5424397-Ins	p.Cys191Ilefs*8, LoF fs	0 / 6	0.075	0.021	0.0022	- / ATCC	10785	70 / 170	High-confidence
FAM111A	11:58919420-Del	p.Leu94Hisfs*22, LoF fs	0 / 8	0.011	0.0294	0.0004	ACTTAAGCTC / -	13647	56 / 80	High-confidence
ATM *LC	**11:108205832-SNV**	**p.V2716A, LoF fs, Pathogenic 7x**	2 [2 AD] / 0	**0.002**	**0.0054**	0.105	T / C	2121	25 / 102	High-confidence
MPZL2 *LC	**11:118133799-Del**	**p.Ile24Metfs*22, LoF fs, Pathogenic 2x**	3 / 0	**0.078, 0.38 [AJ]**	**0.1234**	0.055	T / -	2522	20 / 60	High-confidence
GJB6	13:20797557-Del	p.Lys22Argfs*13, LoF fs, Pathogenic 2x, VUS 1x	0 / 4	0.02	0.0449	0.0125	C / -	2199	18 / 45	High-confidence
MLNR	**13:49796269-Del**	**p.Gln334Valfs*3, LoF fs**	9 / 0	**0.181**	**0.3297**	0.0009 [x PY]	TC / -	11271	50 / 150	High-confidence
*TALPID3 *PF*	14:58899157-Del	p.Arg143Lysfs*4, LoF fs, Pathogenic 9x	10 / 4	0.29	0.4773	0.145 [x FHLC]	G / -	5766	16 / 32	High-confidence
MLKL	16:74709591-Del	p.Asp369Glufs*22, LoF fs	3 [3 SC] / 13	0.255	0.4902	0.0034	TCTG / -	28168	60 / 150	High-confidence
MOB3A	19:2078334-Del	p.Phe69_Ile75del, LoF del	2 / 11	0.062	0.0792	0.0036 [x sex, SMK, PY]	AGATGAGGTTG ACGCGGTTAA / -	5122	13 / 45	High-confidence
CCDC105	19:15132683-Del	p.Gln402Argfs, LoF fs	9 [5 AD] / 0	0.325	0.485	0.0009	C / -	5170	25 / 38	High-confidence
*CHEK2 *LC*	22:29083883-**Ins**	c.*2ins, 3' UTR, VUS 1x	2 / 0	0.004	0.0064	0.105	- / G	979	26 / 30	High-confidence
*CHEK2 *LC*	22:29091207-SNV	p.Ser428Phe, Pathogenic 16x	1 / 3	0.047, 1.12 [AJ]	0.007	0.206	G / A	3766	46 / 65	High-confidence
RHBDD3	22:29659844-Del	p.Gly171Alafs*41, LoF fs	7 [4 AD] / 0	0.112	0.088	0.042	C / -	2795	25 / 45	High-confidence
**50 variants, filtered out based on the sequence read depth and pile-up**										
MED8	1:43850143-Ins	p.Asn295Lysfs, LoF fs	10 / 26	0.086	0.065	0.0014	- / T	386	5 / 25	low complexity
OSBPL9	1:52082713-SNV	c.-16G>C, regulatory TFBS, 5' UTR	9 / 1	0.042	0.0319	0.0263	G / C	282	7 / 22	Single strand bias
SYCP1	1:115469134-Ins	p.Gly629Argfs*12, LoF fs	20 / 3	0.048	0.0463	0.0006	- / A	557	3 / 17	low depth
MEX3A	1:156051590-Ins	p.Gly68Argfs*89, LoF fs	10 / 1	0.012	0.0102	0.0072	- / C	157	2-3 / 20	low depth
FASLG	1:172628474-Del	p.Pro53del, In frame del	0 / 7	0.0015	0.0009	0.0025	CCA / -	171	2-3 / 20	low depth
NEB	2:152359324-SNV	p.Q7971*, LoF Stop gain	9 / 0	0.0019	0.0009	0.0007	G / T	334	10 / 45	low complexity
SRGAP3	3:9027284-Del	p.Ser1074del, In frame del	29 / 10	0.0125	0.0122	0.0094	GCT / -	2183	3-5 / 25	mismatch error
OGG1	3:9816129-SNV	upstream	11 / 1	na	na	0.0038	G / T	361	12 / 40	low complexity
DNAH12	3:57509313-Del	p.Gly93Aspfs*7, LoF fs	8 / 19	0.095	0.0946	0.0091	T / -	449	4 / 20	mismatch
UBA3	3:69112655-SNV	Splice acceptor, LoF	5 / 0	0.006	0	0.0073	C / T	56	4 / 25	low complexity
GYG1	3:148727065-Del	p.Asp163Thrfs*5, LoF fs, Pathogenic 3x	0 / 3	0.077	0.0736	0.0796	G / -	1456	20 / 44	Single strand bias
DGKG	3:185975497-SNV	Noncoding TSE, 17th/24 intron	8 / 0	na	na	0.0092	C / A	446	1-3 / 16	low depth
CRIPAK	4:1388788-snv	p.Asp163Asp	19 / 1	0.0081	0.0459	0.00004	C / T	11686	12 / 80	low complexity
BEND4	4:42145546-Del	p.Glu317del, In frame del	1 / 10	0.0142	0.0139	0.0014	TCC / -	985	8 / 68	mismatch

FER1	5:108168480-SNV	p.Leu73Phe	6 / 0	na	na	0.0079	C / T	152	7 / 36	low complexity
TNFAIP8	5:118704106-SNV	c.31 regulatory TFBS	11 / 2	0.0008	na	0.0108	A / T	207	13 / 40	low complexity
SOX4	6:21595429-Del	p.Gly227del, In frame del	24 / 6	0	0	0.0031	GGC / -	2430	2-4 / 25	mismatch
SP8	7:20824955-Ins	p.Gly161ins, LoF In frame ins	0 / 7	0.0034	0.0031	0.0077	- / CGGACA	1053	0 / 20	mismatch
WBSCR27	7:73254452-Del	Splice acceptor, LoF del	15 / 3	0.0622	0.0595	0.0081	TG / -	5035	2 / 25	low complexity
KMT2E	7:104754096-SNV	3' UTR EncoDE expression	2 / 6	na	na	0.1044	A / T	346	6 / 15	low complexity
CCT4P1	7:140702225-SNV	Downstream	0 / 12	na	na	0.0018	A / T	562	2 / 30	low depth
*CHRNA6 *SMK*	8:42646503-SNV	5' UTR	2 / 12	na	na	0.0039	A / C	1391	3 / 10	low depth
HOOK3	8:42868488-SNV	p.Thr654Lys	13 / 3	na	na	0.0212	C / A	434	6 / 40	Single strand bias
C8orf34	8:69699853-SNV	Noncoding TSE, 12th/13 intron	2 / 13	na	na	0.0023	T / A	109	12 / 40	Single strand bias
CRB2	9:126135888-Ins	p.Gly1036Alafs*43, LoF fs, Pathogenic 3x	1 / 5	0.1509	0.1511	0.1579	- / GGCGCGG CCCCGGCCC	4508	12 / 60	low complexity
*CDKN2A *LC*	9:21978118-SNV	NMD, 4th/5 intron	4 / 13	0.0011	0.0013	0.0135	T / G	2128	4 / 15	low complexity
*CDKN2B *LC*	9:22077884-SNV	Downstream TSE noncoding	16 / 3	na	na	0.0048	A / C	724	20 / 120	Single strand bias
KYAT1	9:131602916-SNV	TFBS-clustered region, 3rd/12 intron	5 / 21	0.0033	0.0034	0.00001	C / A	773	10 / 45	Single strand bias
KMT2D	12:49427266-Ins	p.Gln3745_His3746, In frame Ins	15 / 3	0.195	0.191	0.0061	- / TGC	8761	5 / 40	low complexity
SLCO1B1	12:21327571-SNV	Stop gain p.L96*, LoF	2 / 9	na	na	0.0189	T / G	237	6 / 20	Single strand bias
CMAS	12:22215212-SNV	Splice acceptor, LoF	7 / 0	na	na	0.0036	A / T	292	6 / 52	Single strand bias
*GPC5 *LC*	13:93404337-SNV	3' UTR	15 / 31	na	na	0.0032	G / A	1552	7 / 35	Single strand bias
SNX29	16:12145704-SNV	p.Asp250Val, Splice Acceptor, LoF	6 / 15	0.0012	0.00095	0.0178	A / T	811	7 / 50	low complexity
FBXO47	17:37094756-SNV	3' UTR	8 / 0	na	na	0.0295	A / T	200	5 / 32	Single strand bias
HDAC5	17:42170176-SNV	Splice acceptor, LoF	12 / 2	0.0213	0.019	0.0054	T / G	2495	6 / 22	low complexity
FOXK2	17:80544055-Del	Splice donor, LoF	0 / 7	0.0177	0.019	0.0061	GGAGACCACA / -	700	0 / 25	mapping error
DAZAP1	19:1434830-Del	p.Gly383Alafs*46, LoF fs	3 / 12	0.0102	0.011	0.0065	G / -	95	2-4 / 30	low quality
SAFB2	19:5590343-Del	p.Gly824Alafs*8, LoF fs	11 / 1	0	0	0.0039	C / -	52	2 / 15	low depth
PRKCSH	19:11553290-Del	p.Glu189Glyfs*27, LoF fs	0 / 6	0.0013	0.001	0.0097	AG / -	102	2-3 / 19	low depth
ZNF433	19:12128629-SNV	Downstream TSE	1 / 13	0.0072	0.0075	0.0019	C / A	2004	4 / 17	Single strand bias
mir-1199	19:14185237-Ins	p.Arg346Profs*29, LoF fs	0 / 6	0.0083	0.0087	0.0141	- / C	221	4 / 18	low depth
NPHS1	19:36322581-Del	p.Val1084Serfs, LoF fs, Pathogenic 3x	6 / 2	0.0069	0.0066	0.250	C / -	407	2 / 22	low depth
CCDC97	19:41825698-Del	p.Glu245del In frame del	2 / 11	0.0028	0.0031	0.0037	AGG / -	397	2 / 25	low depth
KCNN4	19:44280866-SNV	TFBS-clustered region, 1st intron	15 / 2	na	na	0.0020	A / C	282	3 / 14	low depth
NOVA2	19:46443628-Del	p.Ala324del, In frame del	18 / 2	0.027	0.021	0.0005	GGC / -	609	3 / 20	low depth
NKX2-4	20:21377790-Del	p.Ala91del, In frame del	3 / 13	0.0177	0.018	0.0033	GCC / -	638	2 / 12	low depth
ASXL1	20:31022442-Ins	p.Gly646Trpfs, LoF fs, Pathogenic 2x	9 / 3	0.0641	0.0636	0.1493	- / G	1714	2 / 25	low depth
RBM10	X:47030467-Del	p.Arg85del, In frame del	20 / 3	0.0035	0.0023	0.0005	GGC / -	981	2-3 / 15	low depth
PLS3	X:114871146-SNV	Splice acceptor, LoF	5 / 0	0	0	0.0063	A / T	103	2-5 / 22	low depth
TEX28P1	X:153482737-SNV	regulatory 5' UTR	15 / 3	0.279	0.24	0.0082	C / T	1073	10 / 20	Single strand bias

Abbreviation: TRICL, Transdisciplinary Research in Cancer of the Lung; gnomAD, the Genome Aggregation Database; Indels, insertion (ins) / deletion (del); SNV, single nucleotide variants; LC, lung cancer; AD, adenocarcinoma; SC, squamous cell carcinoma; SMK, smoking; PF, pulmonary function; LoF, loss of function; fs, frameshift; VUS, variant of uncertain significance; MAF, minor allele frequency; NFE, non-Finnish European; AJ, Ashkenazi Jewish; FHLC, family history of LC; PY, pack-year; UTR, untranslated region; TFBS, transcription factor binding site; TSE, transcription end site; NMD, Nonsense-mediated mRNA decay.

* Known GWAS susceptibility locus in LC, SMK, and PF.

# Number of specific histology (AD or SC) were noted if variant carriers were enriched in a specific sub-histology group.

[&] MAF% from the gnomAD release 2, shown for selected 134,187 non-cancer controls and 64,603 NFE ancestry, respectively.

[†] Epigenetic features (such as TFBS, TSE, NMD) were predicted by the ENCODE project.

[X] Fisher exact test was used for the association analysis. False discovery rate (FDR) adjusted *P*-values were reported. Significant G x E interaction (sex, SMK, PY, FHLC) using a mixed linear regression model.

Supplementary Table 2. Joint analysis of dose-effect and carrier characteristics in the TRICL discovery set

**N. variant alleles of the 25 candidates**	**Dose effect: LC case / Control**		**Characteristics differences between carriers and non-carriers: LC case / Control**							
	N. carriers	OR (95%CI) *	White †	Mean age	Early-onset	Male	FHLC, yes	Smoker	Mean PY	Histology AD
2 protective variants	0 / 6	0.02 (0.01-0.56)	- / 100%	- / 54	- / 83%	- / 83%	- / 17%	- / 67%	- / 20	- / -
1 protective variant	15 / 68	0.19 (0.11-0.33)	93% / 94%	69 / 61	13% / 34%	40% / 60%	47% / 4%	80% / 68%	35 / 15	40% / -
**0 candidate variants**	**922 / 799**	**Reference**	**87% / 94%**	**63 / 62**	**40% / 40%**	**59% / 58%**	**49% / 8%**	**88% / 65%**	**41/ 13**	**44% / -**
1 risk variant	103 / 12	7.54 (4.40-13.71)	90% / 100%	62 / 61	40% / 33%	60% / 47%	45% / 6%	89% / 54%	36 / 11	48% / -
2 risk variants	5 / 0	22.02 (0.51-96*)	100% / -	54 / -	60% / -	20% / -	40% / -	60% / -	22 / -	80% / -
Total	1045 / 885	P trend < 1.0×10^{-8}	87% / 94%	63 / 61	40% / 40%	59% / 58%	48% / 8%	88% / 65%	42 / 23	44% / -

Abbreviations: LC, lung cancer; early-onset (age < 60 yr); OR, odds ratios; FHLC, family history of LC (first degree); PY, pack-year; AD, adenocarcinoma.

* The dose-effect corresponding to increasing numbers of 25 candidates (16 risks and 9 protectives). We set subjects with zero risk and protective variants as the reference group, OR = 1.

† None of the variant allele carriers were from the African population.

Supplementary Table 3. Additional rare deleterious variants in top candidate genes in the TRICL study

Chr position GRCh37/hg19	RS ID	Ref/Alt	HGVS, exon number	ClinVar Interpretations	MAF% & gnomAD	MAF% LC / Control	n. carriers LC / Control	Association *P*-value #
ATM								
11:108121432-SNV	rs866521873	C/A	**Stop-gain** p.Gln414*, exon 10	**Pathogenic** 3 x, Likely benign	**0 &**	0.39 / 0.17	8 / 3	0.2156
11:108160516-SNV	rs34640941	A/G	p.Tyr1475Cys, exon 29	Likely benign 6 x , VUS 4 x	0.058	0.24 / 0.11	5 / 2	0.3589
11:108164137-SNV	rs140856217	T/C	p.Val1570Ala, exon 31	Benign (likely) 5 x, VUS 7 x	0.041	0.19 / 0	4 / 0	**0.0656**
11:108175463-SNV	rs1801673	A/T	p.Asp1853Val, exon 37	Benign (likely) 11 x, VUS 1x	0.488	0.62 / 0.4	13 / 7	0.3287
**11:108205832-SNV Candidate**	**rs587782652**	**T/C**	**p.Val2716Ala, exon 55**	**Pathogenic 7 x**	**0.002**	**0.09 / 0**	**2 / 0**	**0.105**
MPZL2								
**11:118133799-Del Candidate**	**rs752672077**	**T/-**	**p.Ile24fs, LoF del**	**Pathogenic 2 x**	**0.077**	**0.14 / 0**	**3 / 0**	**0.055**
11:118134867-SNV	rs371798268	A/G	**Start-loss**, p.Met1Thr, exon 1	-	0.0131	0.10 / 0	2 / 0	0.1932
POMC								
**2:25383922-Del Candidate**	**rs756770132**	**A/-**	**c.*28delT, miRNAs targets, 3'UTR**	**VUS 2 x**	**0.086**	**0.29 / 0**	**6 / 0**	**0.0066**
2:25384324-SNV	rs201408477	A/G	p.Phe144Leu, exon 3	VUS	0.0168	0.05 / 0	1 / 0	0.3574
2:25384325-SNV	rs201519174	G/C	p.His143Gln, exon 3	VUS	0.0179	0.05 / 0	1 / 0	0.3574
2:25384503-SNV	rs781244602	C/T	**Stop-gain** p.Trp84*, exon 3	**Pathogenic**	**0.0007**	0.05 / 0	1 / 0	0.3574
2:25384578-SNV	rs752644128	G/A	p.Pro59Leu, exon 3	-	0.0057	0.10 / 0	2 / 0	0.1932

Abbreviation: TRICL, Transdisciplinary Research in Cancer of the Lung; LC, lung cancer; SNV, single nucleotide variants; Indels, insertion (ins) / deletion (del); LoF, loss of function; fs, frameshift; VUS, variant of uncertain significance; MAF, minor allele frequency; gnomAD, genome aggregation database.

& MAF% from the gnomAD non-cancer controls (n = 134,187). *ATM* rs866521873 p.Gln414* has been classified as Pathogenic in ClinVar. It is not present in population databases (no frequency in GnomAD).

Fisher exact test was used for the allelic association analysis. FDR adjusted *P*-values were reported.

Supplementary Table 4. Pairwise linkage disequilibrium (LD) analysis of rare variants in *ATM* and *POMC*

Gene	D' (r) statistic	rs34640941	rs140856217	rs1801673	Candidate rs587782652
ATM	rs866521873	0.03 (0.02)	0.05 (0.03)	0.02 (0.01)	0.14 (0.06)
	rs34640941	.	0.05 (0.04)	0.03(0.02)	0.14 (0.08)
	rs140856217	.	.	0.05 (0.02)	0.15 (0.10)
	rs1801673	.	.	.	0.14 (0.04)
POMC	**D' (r) statistic**	rs201519174	rs781244602	rs752644128	**Candidate rs756770132**
	rs201408477	0.22 (0.22)	0.22 (0.22)	0.22 (0.16)	0.22 (0.09)
	rs201519174	.	0.22 (0.22)	0.22 (0.16)	0.22 (0.09)
	rs781244602	.	.	0.22 (0.16)	0.22 (0.09)
	rs752644128	.	.	.	0.14 (0.08)

Supplementary Table 5. The protein-protein interaction network and pathway analyses, predicted by STRING (Search Tool for the Retrieval of Interacting Genes)

**Protein (node 1)**	**Protein (node 2)**	**Interaction score – evidence confidence**
**ATM**	**CHEK2**	**0.999 – highest confidence**
**TP63**	**CHEK2**	**0.71 – high confidence**
**ATM**	**TP63**	**0.57 – medium confidence**
MME	POMC	0.36 – low confidence
ATM	CRB2	0.29 – low confidence
MLNR	POMC	0.25 – low confidence
ATM	PHF13	0.24 – low confidence
CHEK2	CRB2	0.23 – low confidence
CHEK2	POMC	0.19 – low confidence
ATM	POMC	0.19 – low confidence
BMP8A	MLKL	0.18 – low confidence
MME	TP63	0.18 – low confidence
NPHP3	PHF13	0.16 – low confidence
VCPIP1	RHBDD3	0.16 – low confidence
ATM	MME	0.15 – low confidence
**Biological Process (GO) Pathway**	**Number of genes in the network**	**FDR *P*-value**
Replicative senescence	4 of 13	0.0002
Cell aging	5 of 66	0.0005
Cellular response to gamma radiation	4 of 55	0.0067
Mitotic G1 DNA damage checkpoint	3 of 69	0.0226
DNA damage response, signal transduction by p53 class mediator	3 of 82	0.0246
**Kyoto Encyclopedia of Genes and Genomes (KEGG) Pathways**	**Number of genes in the network**	**FDR *P*-value**
Small cell lung cancer	7 of 92	0.0082
Viral carcinogenesis	8 of 183	0.0123
Human T- cell leukemia virus, type 1 infection	9 of 250	0.0123
Non-small cell lung cancer	5 of 66	0.013
Chronic myeloid leukemia	5 of 76	0.0136
